# Inclusion of multiple high‐risk histopathological criteria improves the prediction of adjuvant chemotherapy efficacy in lung adenocarcinoma

**DOI:** 10.1111/his.14301

**Published:** 2021-02-07

**Authors:** Marco Sereno, Zhangyi He, Claire R Smith, Juvenal Baena, Madhumita Das, Robert K Hastings, Grace Rake, Dean A Fennell, Apostolos Nakas, David A Moore, John Le Quesne

**Affiliations:** ^1^ Leicester Cancer Research Centre University of Leicester Leicester UK; ^2^ MRC Toxicology Unit University of Cambridge Leicester UK; ^3^ University Hospitals of Leicester NHS Trust Leicester UK; ^4^ UCL Cancer Institute University College London London UK

**Keywords:** adenocarcinoma of lung, adjuvant, biomarkers, chemotherapy, histology, tumour

## Abstract

**Aims:**

The decision to consider adjuvant chemotherapy (AC) for non‐small cell lung cancer is currently governed by clinical stage. This study aims to assess other routinely collected pathological variables related to metastasis and survival for their ability to predict the efficacy of AC in lung adenocarcinoma.

**Methods and results:**

A retrospective single‐centre series of 620 resected lung non‐mucinous adenocarcinoma cases from 2005 to 2015 was used. Digital images of all slides were subjected to central review, and data on tumour histopathology, AC treatment and patient survival were compiled. A statistical case matching approach was used to counter selection bias. Several high‐risk pathological criteria predict both pathological nodal involvement and early death: positive vascular invasion status (VI+) (HR = 2.10, *P* < 0.001), positive visceral pleural invasion status (VPI+) (HR = 2.16, *P* < 0.001), and solid/micropapillary‐predominant WHO tumour type (SPA/MPPA) (HR = 3.29, *P* < 0.001). Crucially, these criteria also identify patient groups benefiting from AC (VI + HR = 0.69, *P* = 0.167, VPI + HR = 0.44, *P* = 0.004, SPA/MPPA HR = 0.36, *P* = 0.006). Cases showing VI+/VPI+/SPA/MPPA histology in the absence of AC stage criteria were common (170 of 620 total), and 8 had actually received AC. This group showed much better outcomes than equivalent untreated cases in matched analysis (3‐year OS 100.0% versus 31.3%). Inclusion of patients with VI+/VPI+/SPA/MPPA histology would increase AC‐eligible patients from 51.0% to 84.0% of non‐mucinous tumours in our cohort.

**Conclusions:**

Our data provide preliminary evidence that the consideration of AC in patients with additional high‐risk pathological indicators may significantly improve outcomes in operable lung adenocarcinoma, and that AC may be currently underused.

## Graphical abstract

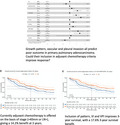

## Introduction

Adjuvant chemotherapy (AC) is now a well‐established component of therapy for non‐small cell lung cancer (NSCLC). Meta‐analyses of prospective studies show an overall patient benefit of 4–5% in 5‐year survival,^1^, ^2^ and current UK and US guidelines recommend AC in cases of clinical stage IIA or above, equivalent to TNM 8th pathology staging of at least pT2b (i.e. >40 mm) and/or positive nodal metastasis.

The biological rationale underlying the efficacy of AC is the elimination of clinically occult metastatic tumour cells which lies beyond the surgically resected field. Nodal involvement and tumour size are clearly key measures of occult metastatic potential, as nodal involvement is direct evidence of metastasis, and for any given invasive tumour any increase in tumour volume offers additional opportunities for tumour seeding. However, several other routinely collected histological data would be expected *a priori* to provide further valuable information. Vascular invasion (VI), visceral pleural invasion (VPI), and histological growth pattern are all good candidate additional biomarkers of benefit. VI directly demonstrates seeding into haematogenous or lymphovascular systems. VPI shows the ability of a tumour to penetrate through the pleural elastin layers and indicates an elevated risk of seeding into the pleural cavity. In adenocarcinoma, areas of *in situ* growth pattern are by definition probably incapable of metastasis, whereas all invasive patterns would be expected to carry this risk, and high‐risk growth patterns are known to be especially likely to recur. Therefore, we set out to determine whether these three histopathological variables, show any ability to predict beneficial effects of AC in a large retrospective case series of resected primary lung adenocarcinomas.

## Material and methods

### Retrospective Cohort of Lung Adenocarcinoma Cases

An electronic database search of the histopathology department within the University Hospitals of Leicester (UHL) NHS Trust was conducted to identify all patients who underwent surgery with curative intent for non‐mucinous primary lung adenocarcinoma from 2005 to 2015. Patients who had been diagnosed with any other lung tumour in the previous 5 years prior to diagnosis were excluded, as were tumours with non‐adenocarcinomatous elements. The complete cohort consisted of 620 cases, of which 516 had complete data in overall stage, T stage, N stage, VI status, VPI status and WHO subtype. These are summarised in Table 1.

**Table 1:** Patient, tumour and adjuvant chemotherapy treatment characteristics

	Treated cases (*n* = 95)	Untreated cases (*n* = 488)	All cases (*n* = 620)
Age at surgery (years)			
*n* (%)	95 (100.0)	488 (100.0)	620 (100.0)
mean (95% CI)	63.5 (49.7, 77.0)	69.2 (54.4, 82.0)	68.1 (52.0, 81.1)
Sex			
Male	26 (27.4)	230 (47.1)	273 (44.0)
Female	69 (72.6)	258 (52.9)	347 (56.0)
Performance status			
0	65 (68.4)	220 (45.1)	299 (48.2)
1	23 (24.2)	198 (40.6)	240 (38.7)
2	7 (7.4)	59 (12.1)	68 (11.0)
3	0 (0.0)	7 (1.4)	8 (1.3)
4	0 (0.0)	1 (0.2)	1 (0.2)
NA	0 (0.0)	3 (0.6)	4 (0.6)
Stage			
0	0 (0.0)	4 (0.8)	4 (0.6)
I	8 (8.4)	240 (49.2)	260 (41.9)
II	29 (30.5)	92 (18.9)	127 (20.5)
III	51 (53.7)	83 (17.0)	148 (23.9)
NA	7 (7.4)	69 (14.1)	81 (13.1)
Tumour size (mm)			
*n* (%)	94 (98.9)	471 (96.5)	601 (96.9)
mean (95% CI)	39.2 (14.0, 80.0)	32.3 (10.0, 70.0)	34.1 (10.0, 72.0)
T stage			
T1	20 (21.0)	195 (40.0)	225 (36.3)
T2	44 (46.3)	160 (32.8)	218 (35.2)
T3	17 (17.9)	76 (15.6)	97 (15.6)
T4	11 (11.6)	30 (6.1)	49 (7.9)
NA	3 (3.2)	27 (5.5)	31 (5.0)
N stage			
N0	26 (27.4)	326 (66.8)	370 (59.7)
N1	24 (25.3)	58 (11.8)	89 (14.3)
N2	51 (43.1)	52 (10.7)	101 (16.3)
NA	4 (4.2)	52 (10.7)	60 (9.7)
Vascular invasion			
Negative	42 (44.2)	260 (53.3)	319 (51.4)
Positive	53 (55.8)	225 (46.1)	298 (48.1)
NA	0 (0.0)	3 (0.6)	3 (0.5)
Pleural invasion			
PL0	47 (49.5)	272 (55.8)	338 (54.5)
PL1	31 (32.6)	129 (26.4)	170 (27.4)
PL2	6 (6.3)	26 (5.3)	35 (5.7)
PL3	6 (6.3)	42 (8.6)	49 (7.9)
NA	5 (5.3)	19 (3.9)	28 (4.5)
WHO subtype			
LPA	8 (8.4)	63 (12.9)	73 (11.8)
APA	44 (46.3)	193 (39.6)	249 (40.2)
PPA	14 (14.7)	70 (14.3)	89 (14.4)
MPPA	1 (1.1)	11 (2.3)	14 (2.3)
SPA	27 (28.4)	128 (26.2)	169 (27.3)
MIA/AIS	1 (1.1)	23 (4.7)	26 (4.0)

LPA, Lepidic adenocarcinoma; APA, Acinar adenocarcinoma; PPA, Papillary adenocarcinoma; MPPA, Micropapillary adenocarcinoma; SPA, Solid adenocarcinoma; MIA, Minimally invasive adenocarcinoma; AIS, Adenocarcinoma *in situ*; NA, Not applicable.

### Clinicopathological Data

Clinical and survival data were collected from pathology reports, local treatment databases, patient records and Public Health England. All tissue and data were collected under NHS ethics agreement 14/EM/1159 approved on 18th September 2014 by the Northampton committee of the National Research Ethics Service.

40× whole section digital images of all tumour slides were obtained using a Hamamatsu nanozoomer XR instrument and reviewed by an experienced subspecialty pathologist (JLQ) to standardise microscopic histological measures. In many cases diagnostic elastin‐stained slides were available for assessment and these data were incorporated into H&E‐based assessment of pleural and vascular invasion. Cases of doubt were examined by and discussed with a second subspecialty pathologist (DM) to obtain a consensus. All positively identified VI was recorded, and no attempt was made to separate haematovascular from lymphovascular invasion. VPI was assessed by invasion of H&E‐stained slide images and was recorded as PL0–PL3 as originally described by Hammar *et al*.^3^ and subsequently incorporated into TNM 7th.^4^ Histopathological staging was performed according to TNM 8th. Tumours were classified by type according to WHO criteria^5^: Adenocarcinoma *in situ* (AIS), minimally invasive adenocarcinoma (MIA), lepidic‐predominant adenocarcinoma (LPA), acinar‐predominant adenocarcinoma (APA), papillary‐predominant adenocarcinoma (PPA), solid‐predominant adenocarcinoma (SPA) and micropapillary‐predominant adenocarcinoma (MPPA).

Details of the chemotherapy regimens applied are incomplete, but in all cases where it is known (48 of 95 treated cases) patients received a standard platinum doublet therapy. We do not anticipate that many patients, if any, received alternative AC regimens.

To minimise selection bias, a balanced subset of the cohort was created for each model of chemotherapy effects. Each treated case was matched with an untreated case matched as closely as possible for sex, tumour size, nodal stage, VI, VPI, predominant growth pattern and performance status, using optimised smallest average absolute propensity score distance across all the matched pairs.^6^ The matching process successfully reduced most absolute standardised mean differences between treated and untreated groups to less than 0.10, indicating that the treated and untreated groups are nearly balanced after matching.

Survival models were generated by standard Cox proportional hazards models and Kaplan–Meier methods. Proportional hazards assumptions were checked by inspection of log‐log plots and Schoenfeld residual‐based tests for Cox proportional hazards models. Kaplan–Meier curves were compared with the (Mantel–Haenszel) log‐rank test and the Peto & Peto test. Compared to the log‐rank test statistic, the Peto & Peto test statistic gives more weight to earlier events, and is thereby more sensitive to early differences between survival. All statistical analyses were conducted in R (version 4.0.2).

## Results

**Figure 1:**
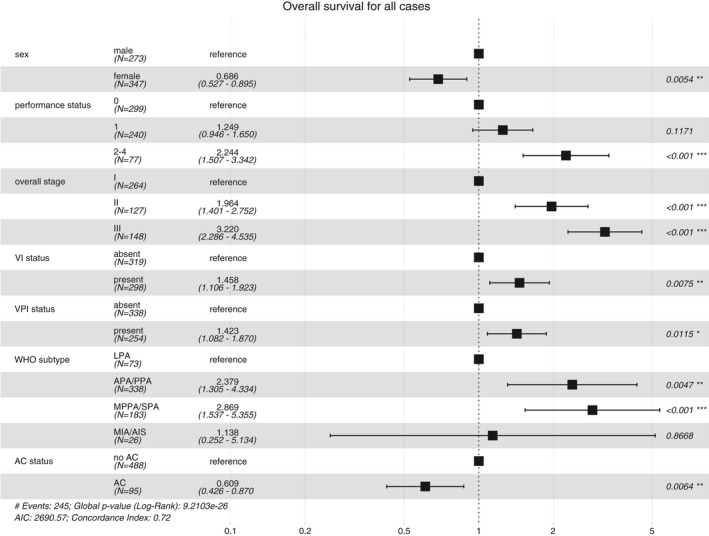
Forest plot showing multivariate Cox model of overall survival by key clinicopathological and clinical variables.

We first established the relationships between key pathological measures and patient survival in our series, both in the entire cohort (Figure S1; Table S1) and in cases not receiving AC (Figure S2; Table S2). As expected, overall pathological stage is strongly related to survival in both groups. We went on to examine three more pathological measures of disease aggressiveness: VI, VPI, and predominant growth pattern. All strongly predict outcome in both univariate (Figures S1B–D and S2B–D; Tables S1 and S2) and multivariate analyses (Figure 1). Furthermore, while VI status, VPI status and predominant growth pattern are all significantly associated with tumour size and pathological lymph node involvement (Table S3), the association between these variables is very weak (Table S4), implying that these three variables convey distinct information as measures of occult metastatic risk.

The degree of VPI shows a clear incremental relationship with poor outcome (Figures S3C and S4C), and cases were divided into VPI+ (i.e. PL1–PL3) versus VPI− cases (i.e. PL0). WHO tumour types were grouped into precursor (MIA/AIS), low‐risk (LPA), moderate‐risk (APA/PPA), and high‐risk (SPA/MPPA) groups (Figures S1D and S2D). This subdivision appears natural and has been previously used in other large studies.^7^, ^8^, ^9^

Given the strength of the links between these variables, metastatic potential and patient survival, we went on to examine their potential as biomarkers of AC efficacy. This was assessed by the construction of survival models to test the effect of chemotherapy in subgroups of our cohort. These analyses were further optimised by selection of matched sets of treated and untreated cases to eliminate treatment selection bias.

**Figure 2:**
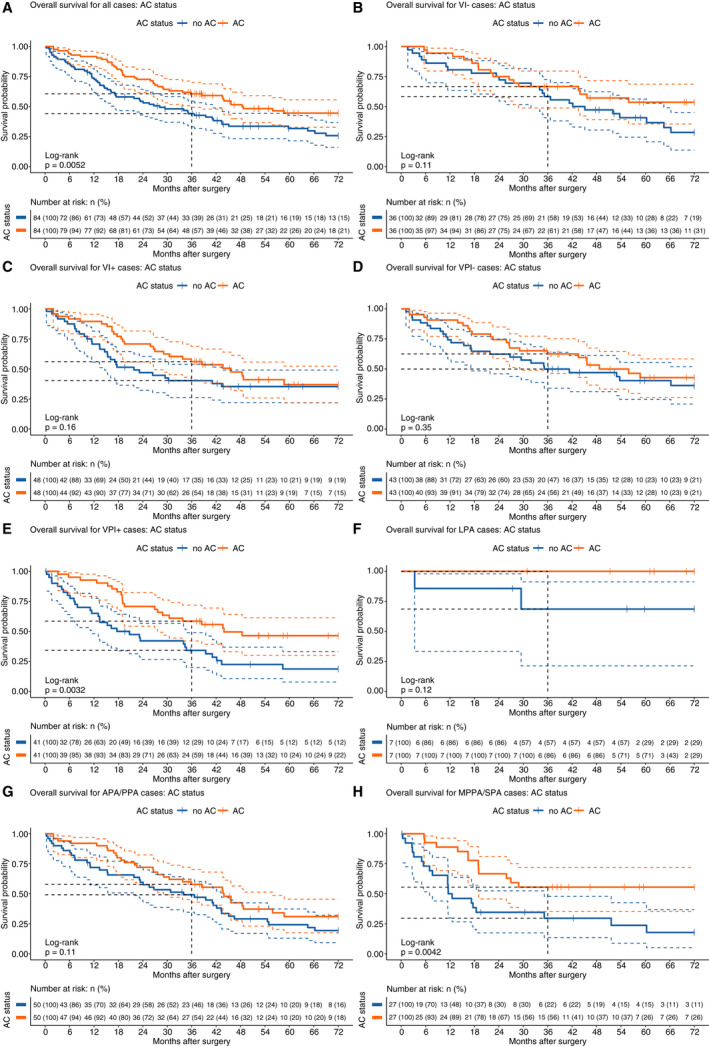
Kaplan–Meier analyses of chemotherapy effects in low‐ and high‐risk pathological subgroups. AC cases were matched with non‐AC cases by propensity score matching. (**A**) All cases, (**B**) vascular invasion negative, (**C**) vascular invasion positive, (**D**) visceral pleural invasion negative (PL0), (**E**) visceral pleural invasion positive (PL1–3), (**F**) low‐risk predominant growth pattern (LPA) (**G**) intermediate‐risk predominant growth patterns (APA/PPA) and (**H**) high‐risk predominant growth patterns (SPA/MPPA).

**Figure 3:**
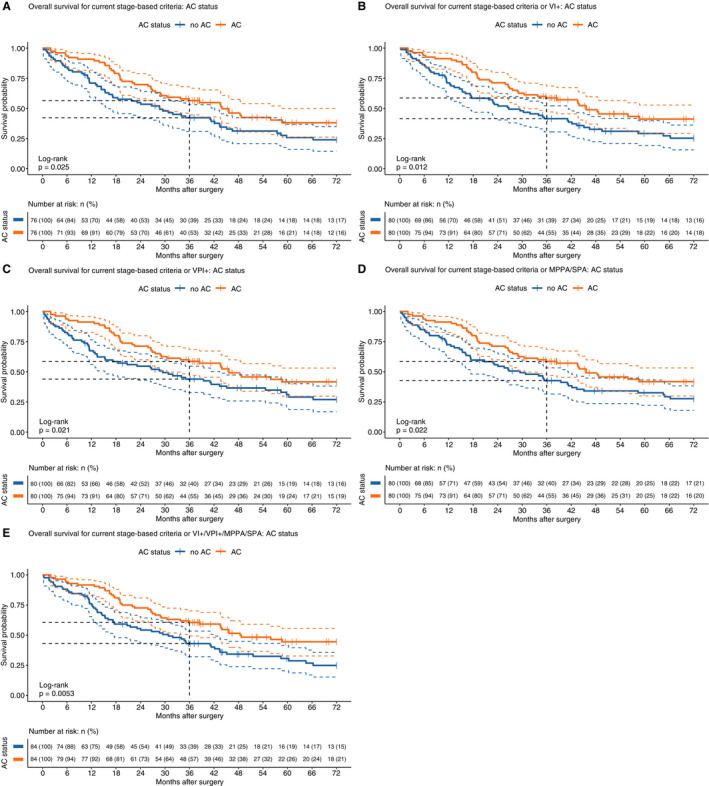
Kaplan–Meier analysis of patient survival comparing AC versus non‐AC outcomes for existing and augmented sets of AC criteria. AC cases were matched with non‐AC cases by propensity score matching. (**A**) Current stage‐based criteria (i.e. overall stage IIA/IIB/IIIA), (**B**) Stage or VI+, (**C**) Stage or VPI+, (**D**) Stage or MPPA/SPPA and (**E**) Stage or VI+/VPI+/SPA/MPPA.

In a matched analysis of all treated cases (Figure 2; Table 2), chemotherapy treatment was related to improved survival (3‐year OS 44.1% versus 60.6% log‐rank *P* = 0.005) (Figure 2A). We then compared the chemotherapy effect in patients with and without histopathological VI (Figure 2B,C). Chemotherapy had no discernible effect in VI− cases but a trend toward beneficial effect of chemotherapy was apparent in VI+ cases at 3 years (Figure 2, 3, 3‐year OS 40.2% versus 56.1% Peto & Peto *P* = 0.053), although significance is lost over 5 years as numbers diminish (Figure 2, 3, 5‐year OS 35.4% versus 37.0% log‐rank *P* = 0.160). Similarly, in cases without evidence of VPI, there was no significant advantage associated with chemotherapy treatment (Figure 2D), while in VPI+ cases there was a significant treatment‐associated benefit (Figure 2, 3, 3‐year OS 34.2% versus 58.5% log‐rank *P* = 0.003). Predominant growth pattern also shows a strong relationship: low‐ and intermediate‐risk groups (LPA/APA/PPA) see no significant benefit (Figure 2F,G), while high‐risk categories (SPA/MPPA) do (Figure 2, 3, 3‐year OS 29.7% versus 55.6% log‐rank *P* = 0.004). Details of all matched patient groups are supplied (Table S5).

**Table 2:** Summary of univariate Cox models for overall survival comparing patients treated with AC versus untreated patients after propensity score matching

Clinicopathological variable	Overall survival after propensity score matching				
	Number of patients (treated versus untreated)	Number of deaths (treated versus untreated)	Hazard ratio	95% CI	*P*‐value
All patients	84 versus 84	43 versus 57	0.572	0.385, 0.851	0.001
Vascular invasion (VI)					
Negative	36 versus 36	16 versus 24	0.604	0.320, 1.137	0.118
Positive	48 versus 48	27 versus 30	0.692	0.411, 1.167	0.167
Visceral pleural invasion (VPI)					
Negative	43 versus 43	22 versus 25	0.761	0.429, 1.350	0.35
Positive	41 versus 41	21 versus 31	0.442	0.253, 0.771	0.004
WHO subtype					
LPA	7 versus 7	0 versus 2	5.075e‐10	0, Inf	0.999
APA/PPA	50 versus 50	31 versus 38	0.683	0.425, 1.098	0.115
SPA/MPPA	27 versus 27	12 versus 20	0.364	0.177, 0.749	0.006

LPA, Lepidic adenocarcinoma; APA, Acinar adenocarcinoma; PPA, Papillary adenocarcinoma; SPA, Solid adenocarcinoma; MPPA, Micropapillary adenocarcinoma.

Unmatched analyses were conducted in parallel (Figure S5; Table S6). No chemotherapy effect was seen in models which include all patients, consistent with selection bias, as patients receiving AC are of more advanced stage. Subgroup analysis, however, shows VI− patients trending toward a poorer outcome with chemotherapy treatment (HR = 1.108, *P* = 0.673) but a better outcome in VI+ patients (HR = 0.753, *P* = 0.151). Similarly, VPI− patients only see a negative effect of chemotherapy (HR = 1.435, *P* = 0.111) while VPI+ patients experience a benefit (HR = 0.539, *P* = 0.007). When analysing groups by predominant pattern, only high‐risk SPA/MPPA categories show a significant trend toward improved survival (HR = 0.552, *P* = 0.046).

Hypothetically, therefore, the inclusion of any or all of these high‐risk features as AC selection criteria might be expected to improve survival outcomes for these patient groups.

In order to test this idea retrospectively, we constructed a further series of matched models to compare the current stage‐based inclusion criteria (i.e. stage IIA/IIB/IIIA) and alternative expanded criteria which included additional high‐risk patient categories. Each model compared two groups of patients: those who met model criteria and received chemotherapy, and those who met model criteria and did not receive chemotherapy (Figure 3). Thus, these models assess treatment efficacy within different subgroups of patients. Allotment of patients for chemotherapy is governed by many factors in addition to stage, such as fitness, post‐operative complications and patient choice, so that it is not unusual for patients not to receive adjuvant therapy despite meeting stage criteria.

Figure 3A shows the performance of the current criteria, in which patients receiving AC experience an early benefit (3‐year OS 42.2% versus 56.5% Peto & Peto *P* = 0.011) although this is attenuated over time (5‐year OS 25.8% versus 38.1% log‐rank *P* = 0.025). The addition of any single additional high‐risk criterion results in a beneficial treatment effect and improves 5‐year survival for AC patients (Figure 3B–D). Inclusion of all three variables has a dramatic effect, showing a 17.6% 3‐year survival benefit for AC‐treated patients, Peto & Peto *P* = 0.003, and 13.8% 5‐year survival benefit for AC‐treated patients, log‐rank *P* = 0.005 (Figure 3E). Equivalent unmatched models were also created in which the benefits of criteria expansion are still apparent in the treated groups, although the cost of withholding treatment is less apparent (Figure S6), suggestive of a treatment selection bias. So, not only do single pathological high‐risk features AC benefit, but their inclusion in AC criteria alongside stage appears to improve survival of treated groups.

What benefit of AC can we discern in the subgroup of patients who show only VI+/VPI+/high‐risk predominant growth pattern but who do not otherwise qualify for AC under current stage‐only criteria? A further model was constructed to test this. 170 patients (159 of which have complete treatment data) meet this description, but unsurprisingly the number who received AC is small (*n* = 8). However, in both matched and unmatched analyses (Figures S7 and S8), this group showed evidence of a highly significant treatment benefit compared to poor prognosis of untreated groups (0 deaths in 8 cases). We were unable to find any additional variables not used for matching which might help to explain the excellent survival of this small group (e.g. younger age, lower smoking history) (Table S7). This preliminary finding illustrates the possibility that a sizeable group of patients who are currently not considered for AC may be being under‐treated. However, given the extremely small size of the group the observation must be treated with caution, especially as it has major implications for therapy. The inclusion of patients displaying these additional high‐risk features in addition to stage would greatly increase the number of non‐mucinous lung cancer cases meeting criteria for AC, increasing the proportion from 51.0% to 84.0% of our cohort (Figure S9).

## Discussion

The introduction of AC into surgical practice for NSCLC has greatly improved patient outcomes. However, compared to other common malignancies, the current stage‐based criteria for the consideration of treatment in lung cancer appear somewhat simplistic. In colon cancer, lymphovascular invasion and poorly differentiated growth are both recommended inclusion criteria in stage II disease.^10^ In breast cancer, multiple tumour features, including proliferation rate and histological grade are routinely integrated alongside stage by decision‐to‐treat tools such as the PREDICT algorithm.^11^ It is plausible that lung cancer patients might also benefit from the inclusion of some high‐risk pathological features, and while some allowance is made for this in the US National Comprehensive Cancer Network (NCCN) guidelines it is supported by lower‐level evidence only.^12^

A previous high‐profile study has addressed the use of predominant growth pattern as a predictor of likely benefit from AC,^13^ finding in a large pooled analysis of published AC trials that high‐risk predominant growth pattern predicts good disease‐specific outcomes. Crucially, our own findings, performed with very different methodology, confirm that high‐risk predominant growth patterns are predictive of good AC effects. Moreover, in addition to high‐risk predominant growth patterns, our data suggest that two other histopathological predictors of nodal metastasis and early death, namely VI+ and VPI+, are also potent predictors of AC benefit.

There is as yet no formal grading scheme in lung adenocarcinoma. VPI is a component of TNM staging, having been adopted for the 7th edition of TNM in 2009^14^ and retained in the 8th edition in 2016.^15^ However, it does not direct high‐risk patients towards AC, as it upstages from T1 to T2a, which in the absence of nodal involvement is stage group IB, and therefore not currently recommended for AC.

In our set of 620 cases, 170 showed the presence of VI+/VPI+/high‐risk predominant growth pattern in the absence of advanced clinical stage. 8 actually received adjuvant chemotherapy; we do not know why, but it is possible that the presence of high‐risk pathological features might have prompted this, despite not being part of current UK guidelines. Suggestively, their survival was very much better than a matched group. This suggests that patients falling into this subgroup may not only benefit from AC, but may be especially likely to benefit, perhaps because metastases derived from these early‐stage tumours are less likely to be well established and are present a greater chance of elimination by AC. Interestingly, previous studies have suggested that earlier‐stage tumours may benefit from AC,^16^ and our findings would suggest that this observation may be driven partly by the presence of additional high‐risk features.

We acknowledge the limitations of our single‐centre retrospective approach: the number of cases overall, and especially of AC cases is relatively small. We would have liked to include recurrence‐free survival data as an additional endpoint, but unfortunately as the data were collected retrospectively, we did not detect enough recurrence events to be powered. There is no true patient randomisation, and we do not know the full details of chemotherapy regimens applied or the decision‐making processes behind them. However, our approach brings the major advantage that it has been possible to collect key pathological data that have not been widely considered for their value as predictive biomarkers in this context. The problem of selection bias was ameliorated by the use of a case matching approach to retrospectively simulate study randomisation. Despite this, and especially given the major increase in the use of adjuvant chemotherapy which our findings might suggest, we stress that these findings are exploratory in nature.

Reassuringly, this study recapitulates several key findings from other studies, such as the independent prognostic value of VI, VPI, and predominant growth pattern, the overall benefit of AC under current selection criteria, and the finding that subtype classification predicts chemotherapy benefit.^13^ Furthermore, the biological rationale for the use of additional high‐risk histopathological features in AC administration is strong. Therefore, we think it reasonable that VI, VPI, and predominant growth pattern may have potential in the future to generally predict AC benefit in lung adenocarcinoma. Furthermore, our results lend additional support to the recommendation in the US NCCN guidelines that high‐risk factors may be considered in the decision to offer AC in resected stage IB/IIA NSCLC.

However, as they stand our observations remain exploratory and preliminary in nature. They should be tested in a suitably designed randomised prospective study with a sound histopathological footing and careful recording of recurrence as an endpoint. The implications in other subtypes of NSCLC, especially squamous cell carcinoma, also need to be examined. If validated, these proposed modifications would be wide‐reaching, suggesting that many additional patients ought to be considered for post‐operative chemotherapy for the best chance of long‐term survival.

## Conflicts of interest

The authors declare that there are no conflicts of interests to disclose.

## Author contributions

MS and CS supported cohort assembly; extensively compiled clinicopathological data for the cohort; maintained the cohort database throughout the study; and performed initial statistical analyses. ZH performed statistical analyses. JB and MD provided expert histological support. RH supported database setup for the study and designed its initial architecture. GR substantively acquired and managed clinical data from patient records. DF, AN, and DM provided critical input into manuscript preparation and cohort assembly. JLQ conceived and oversaw the study and wrote the manuscript.

## Supporting information


**Table S1.** Summary of univariate Cox models for overall survival (all cases).
**Table S2.** Summary of univariate Cox models for overall survival (untreated cases only).
**Table S3.** Pearson's Chi‐squared test of association between each pair of clinicopathological variables.
**Table S4.** Goodman and Kruskal's tau measure of association between each pair of clinicopathological variables.
**Table S5.** Summary demographics for all propensity score matched analysis groups (treated versus non‐treated cases).
**Table S6.** Summary of univariate Cox models for overall survival comparing patients treated with AC versus untreated patients before propensity score matching.
**Table S7.** Demographics and clinicopathological details of the 11 patients treated with AC who show only VI+/VPI+/high‐risk predominant growth pattern but who do not otherwise qualify for AC under current stage‐only criteria.
**Table S8.** Continued: Demographics and clinicopathological details of the 8 treated patients who show only VI+/VPI+/high‐risk predominant growth pattern but who do not otherwise qualify for AC under current stage‐only criteria.
**Figure S1.** Kaplan–Meier analyses of overall survival by simplified pathological subgroups in the entire cohort of 620 patients.
**Figure S2.** Kaplan–Meier analyses of overall survival by simplified pathological subgroups in patients who did not receive AC.
**Figure S3.** Kaplan–Meier analyses of overall survival by detailed pathological subgroups in the entire cohort of 620 patients.
**Figure S4.** Kaplan–Meier analyses of overall survival by detailed pathological subgroups in patients who did not receive AC.
**Figure S5.** Unmatched Kaplan–Meier analyses of chemotherapy effects in low‐ and high‐risk pathological subgroups.
**Figure S6.** Unmatched Kaplan–Meier analysis of patient survival comparing AC‐ versus non‐AC outcomes for existing and augmented sets of AC criteria.
**Figure S7.** Matched Kaplan–Meier analysis of chemotherapy effects in cases showing high‐risk histopathological features (VI+/VPI+/SPA/MPPA) but not meeting current stage‐based criteria.
**Figure S8.** Unmatched Kaplan–Meier analysis of chemotherapy effects in cases showing high‐risk histopathological features (VI+/VPI+/SPA/MPPA) but not meeting current stage‐based criteria.
**Figure S9.** Comparison of the proportion of non‐mucinous lung adenocarcinoma cases meeting existing and augmented criteria for the consideration of AC.
