# Supplementary material for: Inclusion of multiple high‐risk histopathological criteria improves the prediction of adjuvant chemotherapy efficacy in lung adenocarcinoma
Source: Histopathology. 2021 Feb 7;78(6):838–48. doi: 10.1111/his.14301 (PMC8247971; doi:10.1111/his.14301)
Supplement: Supplementary file 1 — Table S1. Summary of univariate Cox models for overall survival (all cases). Table S2. Summary of univariate Cox models for overall survival (untreated cases only). Table S3. Pearson's Chi‐squared test of association between each pair of clinicopathological variables. Table S4. Goodman and Kruskal's tau measure of association between each pair of clinicopathological variables. Table S5. Summary demographics for all propensity score matched analysis groups (treated versus non‐treated cases). Table S6. Summary of univariate Cox models for overall survival comparing patients treated with AC versus untreated patients before propensity score matching. Table S7. Demographics and clinicopathological details of the 11 patients treated with AC who show only VI+/VPI+/high‐risk predominant growth pattern but who do not otherwise qualify for AC under current stage‐only criteria. Table S8. Continued: Demographics and clinicopathological details of the 8 treated patients who show only VI+/VPI+/high‐risk predominant growth pattern but who do not otherwise qualify for AC under current stage‐only criteria. Figure S1. Kaplan–Meier analyses of overall survival by simplified pathological subgroups in the entire cohort of 620 patients. Figure S2. Kaplan–Meier analyses of overall survival by simplified pathological subgroups in patients who did not receive AC. Figure S3. Kaplan–Meier analyses of overall survival by detailed pathological subgroups in the entire cohort of 620 patients. Figure S4. Kaplan–Meier analyses of overall survival by detailed pathological subgroups in patients who did not receive AC. Figure S5. Unmatched Kaplan–Meier analyses of chemotherapy effects in low‐ and high‐risk pathological subgroups. Figure S6. Unmatched Kaplan–Meier analysis of patient survival comparing AC‐ versus non‐AC outcomes for existing and augmented sets of AC criteria. Figure S7. Matched Kaplan–Meier analysis of chemotherapy effects in cases showing high‐risk histopathological features (VI+/VPI+ [file HIS-78-838-s001.docx]

**Inclusion of multiple high-risk histopathological criteria improves the prediction of adjuvant chemotherapy efficacy in lung adenocarcinoma**

**SUPPORTING INFORMATION**

**Contents**

| **Supporting Information** | **Page Number** |
| --- | --- |
| **Table S1:** Summary of univariate Cox models for overall survival (All cases) | 3 |
| **Table S2:** Summary of univariate Cox models for overall survival (Untreated cases only) | 4 |
| **Table S3:** Pearson's Chi-squared test of association between each pair of clinicopathological variables | 5 |
| **Table S4:** Goodman and Kruskal's tau measure of association between each pair of clinicopathological variables | 5 |
| **Table S6:** Summary demographics for all propensity score matched analysis groups (treated vs non-treated cases) | 6 |
| **Table S7:** Summary of univariate Cox models for overall survival comparing patients treated with AC vs. untreated patients before propensity score matching | 8 |
| **Table S8:** Demographics and clinicopathological details of the 11 patients treated with AC who show only VI+/VPI+/high-risk predominant growth pattern but who do not otherwise qualify for AC under current stage-only criteria | 9 |
| **Figure S1:** Kaplan-Meier analyses of overall survival by simplified pathological subgroups in the entire cohort of 620 patients | 11 |
| **Figure S2:** Kaplan-Meier analyses of overall survival by simplified pathological subgroups in patients who did not receive AC | 13 |
| **Figure S3:** Kaplan-Meier analyses of overall survival by detailed pathological subgroups in the entire cohort of 620 patients | 15 |
| **Figure S4:** Kaplan-Meier analyses of overall survival by detailed pathological subgroups in patients who did not receive AC | 17 |
| **Figure S5**: Unmatched Kaplan-Meier analyses of chemotherapy effects in low- and high-risk pathological subgroups | 19 |
| **Figure S6:** Unmatched Kaplan-Meier analyses of patient survival comparing AC- vs non-AC outcomes for existing and augmented sets of AC criteria | 21 |
| **Figure S7:** Matched Kaplan-Meier analysis of chemotherapy effects in cases showing high-risk histopathological features (VI+/VPI+/SPA/MPPA) but not meeting current stage-based criteria. | 24 |
| **Figure S8:** Unmatched Kaplan-Meier analysis of chemotherapy effects in cases showing high-risk histopathological features (VI+/VPI+/SPA/MPPA) but not meeting current stage-based criteria. | 25 |
| **Figure S9:** Comparison of the proportion of non-mucinous lung adenocarcinoma cases meeting existing and augmented criteria for the consideration of AC. | 26 |

**Table S1: Summary of univariate Cox models for overall survival (All cases)**

| **Clinicopathological Variable** | **Number of Patients** | **Number of Deaths** | **Hazard Ratio** | **95% CI** | **P-value** |
| --- | --- | --- | --- | --- | --- |
| **Overall Stage (TNM 8^th^ Edition)**  II vs. I  III vs. I | 539  127 vs. 264  148 vs. 264 | 285  78 vs. 97  110 vs. 97 | 2.075  3.125 | (1.539, 2.796)  (2.375, 4.112) | **<0.001**  **<0.001** |
| **Vascular Invasion (VI)**  Positive vs. Negative | 617  298 vs. 319 | 334  192 vs. 142 | 2.075 | (1.667, 2.583) | **<0.001** |
| **Visceral Pleural Invasion (VPI)**  Positive vs. Negative | 592  254 vs. 338 | 317  175 vs. 142 | 2.156 | (1.727, 2.692) | **<0.001** |
| **WHO Subtype^a^**  APA/PPA vs. LPA  SPA/MPPA vs. LPA  MIA/AIS vs. LPA | 620  338 vs. 73  183 vs. 73  26 vs. 73 | 336  192 vs. 21  117 vs. 21  6 vs. 21 | 2.362  3.285  0.759 | (1.505, 3.706)  (2.064, 5.231)  (0.306, 1.880) | **<0.001**  **<0.001**  0.551 |

**Notes:**

**^a^** - LPA: Lepidic adenocarcinoma; APA: Acinar adenocarcinoma; PPA: Papillary adenocarcinoma; SPA: Solid adenocarcinoma; MPPA: Micropapillary adenocarcinoma; MIA: Minimally invasive adenocarcinoma; AIS: Adenocarcinoma *in-situ*

**Table S2: Summary of univariate Cox models for overall survival (Untreated cases only)**

| **Clinicopathological Variable** | **Number of Patients** | **Number of Deaths** | **Hazard Ratio** | **95% CI** | **P-value** |
| --- | --- | --- | --- | --- | --- |
| **Overall Stage (TNM 8^th^ Edition)**  II vs. I  III vs. I | 419  92 vs. 244  83 vs. 244 | 212  60 vs. 86  66 vs. 86 | 2.409  4.175 | (1.731, 3.352)  (3.021, 5.771) | **<0.001**  **<0.001** |
| **Vascular Invasion (VI)**  Positive vs. Negative | 485  225 vs. 260 | 255  144 vs. 111 | 2.179 | (1.697, 2.798) | **<0.001** |
| **Visceral Pleural Invasion (VPI)**  Positive vs. Negative | 469  197 vs. 272 | 246  142 vs. 104 | 2.618 | (2.031, 3.376) | **<0.001** |
| **WHO Subtype^a^**  APA/PPA vs. LPA  SPA/MPPA vs. LPA  MIA/AIS vs. LPA | 488  263 vs. 63  139 vs. 63  23 vs. 63 | 257  143 vs. 18  91 vs. 18  5 vs. 18 | 2.248  3.451  0.710 | (1.377, 3.671)  (2.080, 5.727)  (0.263, 1.911) | **0.001**  **<0.001**  0.497 |

**Notes:**

**^a^** - LPA: Lepidic adenocarcinoma; APA: Acinar adenocarcinoma; PPA: Papillary adenocarcinoma; SPA: Solid adenocarcinoma; MPPA: Micropapillary adenocarcinoma; MIA: Minimally invasive adenocarcinoma; AIS: Adenocarcinoma *in-situ*

**Table S3: Pearson's Chi-squared test of association between each pair of clinicopathological variables**

|  | **Pearson’s Chi-squared test (P-value)** | | | | | | |
| --- | --- | --- | --- | --- | --- | --- | --- |
|  | | **Overall Stage ^a^** | **Pathological T Stage ^a^** | **Pathological N Stage ^a^** | **VI Status** | **VPI Status** | **WHO Subtype** |
| **Overall Stage ^a^** | | --- | <0.001 | <0.001 | <0.001 | <0.001 | <0.001 |
| **Pathological T Stage ^a^** | | --- | --- | <0.001 | <0.001 | <0.001 | <0.001 |
| **Pathological N Stage ^a^** | | --- | --- | --- | <0.001 | 0.002 | 0.004 |
| **VI Status** | | --- | --- | --- | --- | <0.001 | <0.001 |
| **VPI Status** | | --- | --- | --- | --- | --- | <0.001 |
| **WHO Subtype** | | --- | --- | --- | --- | --- | --- |

**Notes:**

**^a^** – TNM 8^th^ Edition

**Table S4: Goodman and Kruskal's tau measure of association between each pair of clinicopathological variables**

|  | **Goodman and Kruskal's tau measure** | | | | | | |
| --- | --- | --- | --- | --- | --- | --- | --- |
|  | | **Overall Stage ^a^** | **Pathological T Stage ^a^** | **Pathological N Stage ^a^** | **VI Status** | **VPI Status** | **WHO Subtype** |
| **Overall Stage ^a^** | | 4.000 | 0.166 | 0.530 | 0.065 | 0.075 | 0.012 |
| **Pathological T Stage ^a^** | | 0.297 | 5.000 | 0.042 | 0.054 | 0.340 | 0.027 |
| **Pathological N Stage ^a^** | | 0.497 | 0.033 | 4.000 | 0.071 | 0.021 | 0.012 |
| **VI Status** | | 0.027 | 0.019 | 0.029 | 3.000 | 0.036 | 0.028 |
| **VPI Status** | | 0.041 | 0.162 | 0.011 | 0.040 | 3.000 | 0.015 |
| **WHO Subtype** | | 0.021 | 0.039 | 0.020 | 0.073 | 0.052 | 4.000 |

**Notes:**

**^a^** – TNM 8^th^ Edition

**Table S5: Summary demographics for all propensity score matched analysis groups (treated vs non-treated cases)**

| **Clinicopathological Variables** | | | **All cases** | | | | **VI - Cases** | | | | **VI+ Cases** | | | | **VPI- Cases** | | | |
| --- | --- | --- | --- | --- | --- | --- | --- | --- | --- | --- | --- | --- | --- | --- | --- | --- | --- | --- |
|  |  |  | **Treated (*n*=84)** | | **Untreated (*n*=84)** | | **Treated *(n*=36)** | | **Untreated (*n*=36)** | | **Treated (*n*=48)** | | **Untreated (*n*=48)** | | **Treated (*n*=43)** | | **Untreated (*n*=43)** | |
| **Sex** | Male  Female | 24  60 | | 23  61 | | 10  26 | | 15  21 | | 14  34 | | 14  34 | | 9  34 | | 16  27 | |  |
| **Performance Status** | 0  1  2-4 | 57  21  6 | | 50  27  7 | | 28  7  1 | | 28  8  0 | | 29  14  5 | | 25  15  8 | | 29  11  3 | | 26  12  5 | |  |
| **Pathological T Stage ^a^** | T1  T2  T3  T4 | 16  42  17  9 | | 12  43  19  10 | | 6  20  7  3 | | 5  19  8  4 | | 10  22  10  6 | | 10  17  14  7 | | 16  18  6  3 | | 18  19  5  1 | |  |
| **Pathological N Stage ^a^** | N0  N1  N2 | 23  24  37 | | 23  28  33 | | 13  10  13 | | 16  10  10 | | 10  14  24 | | 9  16  23 | | 7  14  22 | | 8  19  16 | |  |
| **Vascular Invasion (VI)** | Absent  Present | 36  48 | | 32  52 | | N/A | | | | N/A | | | | 21  22 | | 19  24 | |  |
| **Visceral Pleural Invasion (VPI)** | Absent  Present | 43  41 | | 36  48 | | 21  15 | | 17  19 | | 22  26 | | 19  29 | | N/A | | | |  |
| **WHO Subtype ^b^** | LPA  APA/PPA  MPPA/SPA | 7  50  27 | | 9  43  32 | | 3  22  11 | | 0  27  9 | | 4  28  16 | | 3  26  19 | | 6  27  10 | | 6  23  14 | |  |

**Notes:**

**^a^** – TNM 8^th^ edition

**^b^** – LPA: Lepidic adenocarcinoma; APA: Acinar adenocarcinoma; PPA: Papillary adenocarcinoma; SPA: Solid adenocarcinoma; MPPA: Micropapillary adenocarcinoma; MIA: Minimally invasive adenocarcinoma; AIS: Adenocarcinoma *in-situ*

**Table S5 Continued: Summary demographics for all propensity score matched analysis groups (treated vs non-treated cases)**

| **Clinicopathological Variables** | | **VPI+ Cases** | | **LPA Cases** | | **APA/PPA Cases** | | **SPA/MPPA Cases** | | |
| --- | --- | --- | --- | --- | --- | --- | --- | --- | --- | --- |
|  |  | **Treated (*n*=41)** | **Untreated (*n*=41)** | **Treated *(n*=7)** | **Untreated (*n*=7)** | **Treated (*n*=50)** | **Untreated (*n*=50)** | **Treated (*n*=27)** | **Untreated (*n*=27)** | |
| **Sex** | Male  Female | 15  26 | 20  21 | 0  7 | 3  4 | 17  33 | 25  25 | 7  20 | 7  20 |  |
| **Performance Status** | 0  1  2-4 | 28  10  3 | 30  8  3 | 4  3  0 | 3  4  0 | 34  14  2 | 30  20  0 | 19  4  4 | 19  4  4 |  |
| **Pathological T Stage ^a^** | T1  T2  T3  T4 | 0  24  11  6 | 0  21  11  9 | 3  2  2  0 | 1  3  3  0 | 11  26  8  5 | 8  22  13  7 | 2  14  7  4 | 1  15  5  6 |  |
| **Pathological N Stage ^a^** | N0  N1  N2 | 16  10  15 | 13  12  16 | 1  3  3 | 5  1  1 | 12  12  26 | 12  18  20 | 10  9  8 | 11  8  8 |  |
| **Vascular Invasion (VI)** | Absent  Present | 15  26 | 13  28 | 3  4 | 5  2 | 22  28 | 22  28 | 11  16 | 8  19 |  |
| **Visceral Pleural Invasion (VPI)** | Absent  Present | N/A | | 6  1 | 4  3 | 27  23 | 19  31 | 10  17 | 8  19 |  |
| **WHO Subtype ^b^** | LPA  APA/PPA  MPPA/SPA | 1  23  17 | 1  24  16 | N/A | | N/A | | N/A | |  |

**Notes:**

**^a^** – TNM 8^th^ edition

**^b^** – LPA: Lepidic adenocarcinoma; APA: Acinar adenocarcinoma; PPA: Papillary adenocarcinoma; SPA: Solid adenocarcinoma; MPPA: Micropapillary adenocarcinoma; MIA: Minimally invasive adenocarcinoma; AIS: Adenocarcinoma *in-situ*

**Table S6: Summary of univariate Cox models for overall survival comparing patients treated with AC vs. untreated patients before propensity score matching**

| **Overall survival before propensity score matching** | | | | | |
| --- | --- | --- | --- | --- | --- |
|  | **Number of patients (Treated *vs.* Untreated)** | **Number of Deaths (Treated *vs.* Untreated)** | **Hazard Ratio** | **95% CI** | **P-value** |
| **All Patients** | 95 vs. 488 | 51 vs. 257 | 0.954 | (0.706, 1.288) | 0.758 |
| **Vascular Invasion (VI)**  Negative  Positive | 42 vs. 260  53 vs. 225 | 20 vs. 111  31 vs. 144 | 1.108  0.753 | (0.688, 1.784)  (0.510, 1.109) | 0.673  0.151 |
| **Visceral Pleural Invasion (VPI)**  Negative  Positive | 47 vs. 272  43 vs. 197 | 24 vs. 104  22 vs. 142 | 1.435  0.539 | (0.920, 2.237)  (0.344, 0.845) | 0.111  **0.007** |
| **WHO Subtype^a^**  LPA  APA/PPA  SPA/MPPA | 8 vs. 63  58 vs. 263  28 vs. 139 | 1 vs. 18  37 vs. 143  13 vs. 91 | 0.381  1.186  0.552 | (0.051, 2.863)  (0.826, 1.703)  (0.309, 0.988) | 0.349  0.356  **0.046** |

**Notes:**

**^a^** – LPA: Lepidic adenocarcinoma; APA: Acinar adenocarcinoma; PPA: Papillary adenocarcinoma; SPA: Solid adenocarcinoma; MPPA: Micropapillary adenocarcinoma

**Table S7:** **Demographics and clinicopathological details of the 8 patients treated with AC who show only VI+/VPI+/high-risk predominant growth pattern but who do not otherwise qualify for AC under current stage-only criteria**

|  | **Overall Survival Status** | **Overall Survival Time (Days)** | **Cancer-Specific Survival Status** | **Cancer-Specific Survival Time (Days)** | **Recurrence-Free Survival Status** | **Recurrence-Free Survival Time (Days)** | **Age at Surgery (Years)** | **Sex** | **Smoking History** | **Performance Status** | **Surgical Procedure** |
| --- | --- | --- | --- | --- | --- | --- | --- | --- | --- | --- | --- |
| **Patient 1** | Alive at last known date | 2191.5 | Alive at last known date | 2191.5 | Recurrence | 781.0 | 67 | Female | Current smoker | 1 | Lobectomy |
| **Patient 2** | Alive at last known date | 1593.0 | Non-cancer related death | 1593.0 | No recurrence | 1593.0 | 73 | Female | Ex-smoker | 0 | Wedge |
| **Patient 3** | Alive at last known date | 1180.0 | Alive at last known date | 1180.0 | No recurrence | 895.0 | 67 | Female | Current smoker | 2 | Lobectomy |
| **Patient 4** | Alive at last known date | 2191.5 | Alive at last known date | 2191.5 | No recurrence | 2191.5 | 54 | Female | Current smoker | 0 | Lobectomy |
| **Patient 5** | Alive at last known date | 2191.5 | Alive at last known date | 2191.5 | No recurrence | 2191.5 | 64 | Male | Ex-smoker | 0 | Lobectomy |
| **Patient 6** | Alive at last known date | 2191.5 | Alive at last known date | 2191.5 | No recurrence | 2191.5 | 64 | Female | Ex-smoker | 0 | Lobectomy |
| **Patient 7** | Alive at last known date | 2191.5 | Alive at last known date | 2191.5 | No recurrence | 2191.5 | 46 | Female | Current smoker | 2 | Lobectomy |
| **Patient 8** | Alive at last known date | 2191.5 | Alive at last known date | 2191.5 | Recurrence | 1064.0 | 50 | Female | Current smoker | 0 | Lobectomy |

**Table S8 Continued: Demographics and clinicopathological details of the 8 treated patients who show only VI+/VPI+/high-risk predominant growth pattern but who do not otherwise qualify for AC under current stage-only criteria**

|  | **Residual Tumour ^a^** | **Tumour Site ^b^** | **Overall Stage ^c^** | **Tumour Size (mm)** | **Pathological T Stage ^c^** | **Pathological N Stage ^c^** | **Vascular Invasion (VI)** | **Visceral Pleural Invasion (VPI)** | **WHO Subtype ^d^** | **Predominant Growth Pattern ^e^** | **Adjuvant Chemotherapy (AC) Status** |
| --- | --- | --- | --- | --- | --- | --- | --- | --- | --- | --- | --- |
| **Patient 1** | R0 | RUL | IA | 28 | T1c | N0 | Present | PL0 | APA | AC | Received AC |
| **Patient 2** | R0 | LUL | IB | 22 | T2a | N0 | Present | PL2 | APA | AC | Received AC |
| **Patient 3** | R0 | LUL | IB | 40 | T2a | N0 | Present | PL0 | APA | AC | Received AC |
| **Patient 4** | R0 | N/A | IB | 39 | T2a | N0 | Absent | PL0 | SPA | SOLID | Received AC |
| **Patient 5** | R0 | RUL | IB | 40 | T2a | N0 | Absent | PL1 | APA | AC | Received AC |
| **Patient 6** | R0 | RLL | IB | 35 | T2a | N0 | Absent | PL0 | SPA | SOLID | Received AC |
| **Patient 7** | R0 | RUL | IB | 27 | T2a | N0 | Present | PL1 | SPA | SOLID | Received AC |
| **Patient 8** | R0 | RUL | IB | 30 | T2a | N0 | Absent | PL1 | SPA | SOLID | Received AC |

**Notes:**

**^a^** – R0: Tumour completely excised at surgery

**^b^** – LUL: Left upper lobe; RLL: Right lower lobe; RUL: Right upper lobe

**^c^** – TNM 8^th^ edition

**^d^** – APA: Acinar adenocarcinoma; SPA: Solid adenocarcinoma

**^e^** – AC: Acinar

**Figure S1: Kaplan-Meier analyses of overall survival by simplified pathological subgroups in the entire cohort of 620 patients**


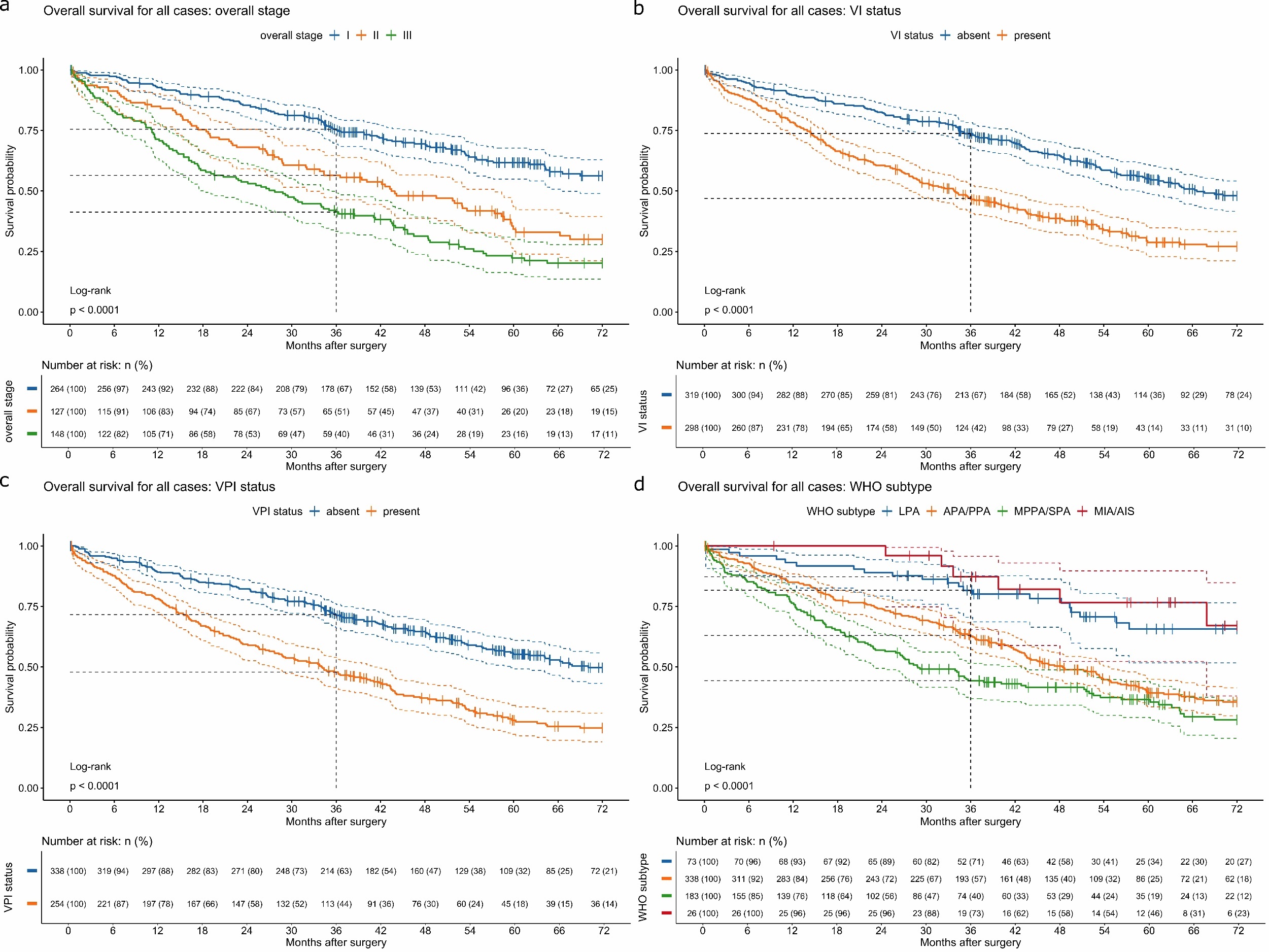


**Notes:**

The patient cohort was divided by a) Overall Stage (TNM 8^th^ edition); b) Vascular invasion status. Significance was assessed by log-rank test.

**Figure S1 Continued: Kaplan-Meier analyses of overall survival by simplified pathological subgroups in the entire cohort of 620 patients**


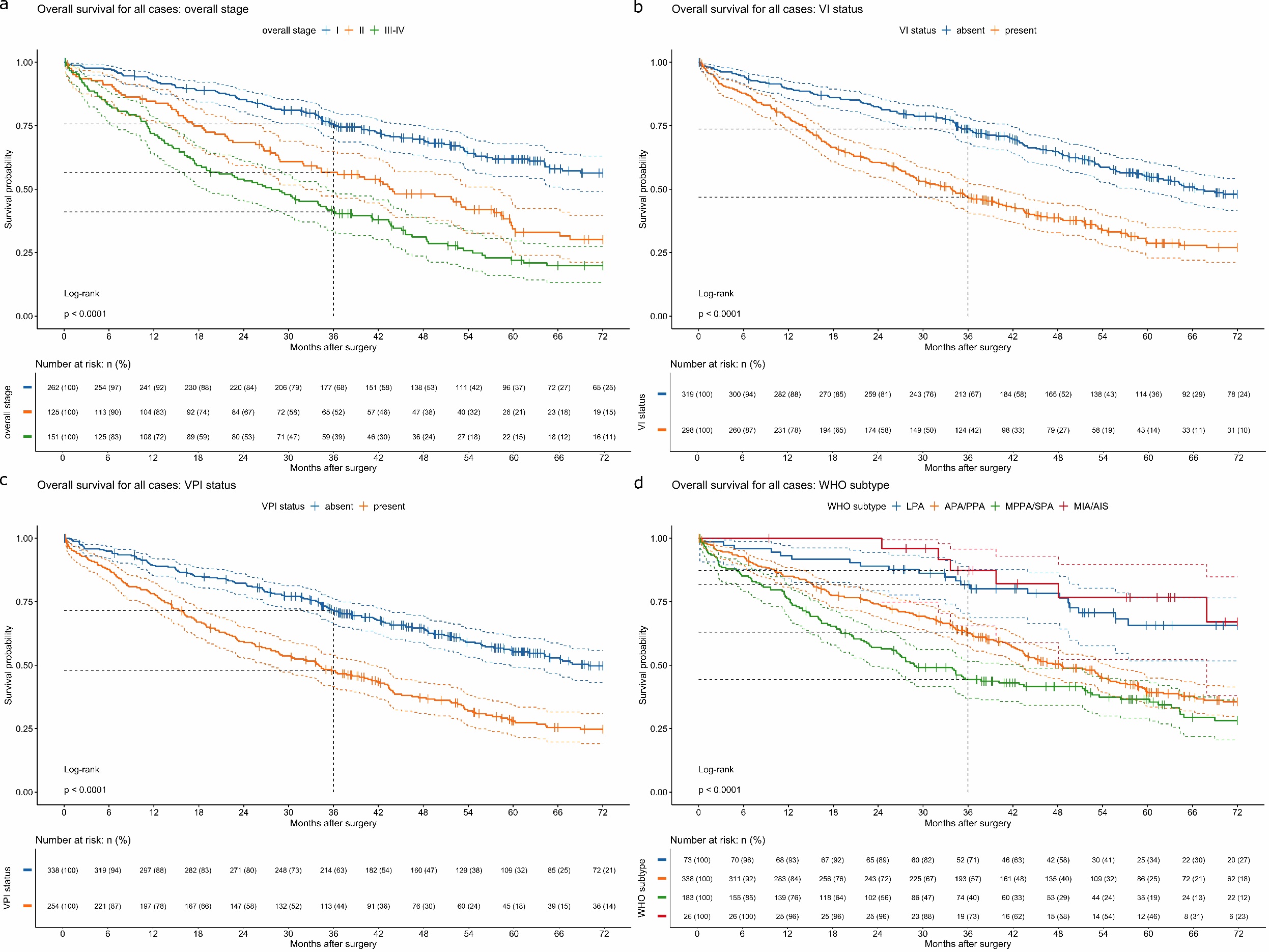


**Notes:**

The patient cohort was divided by c) Visceral pleural invasion status; d) WHO subtype ^a^. Significance was assessed by log-rank test.

**^a^** – LPA: Lepidic adenocarcinoma; APA: Acinar adenocarcinoma; PPA: Papillary adenocarcinoma; SPA: Solid adenocarcinoma; MPPA: Micropapillary adenocarcinoma; MIA: Minimally invasive adenocarcinoma; AIS: Adenocarcinoma *in-situ*

**Figure S2: Kaplan-Meier analyses of overall survival by simplified pathological subgroups in patients who did not receive AC**


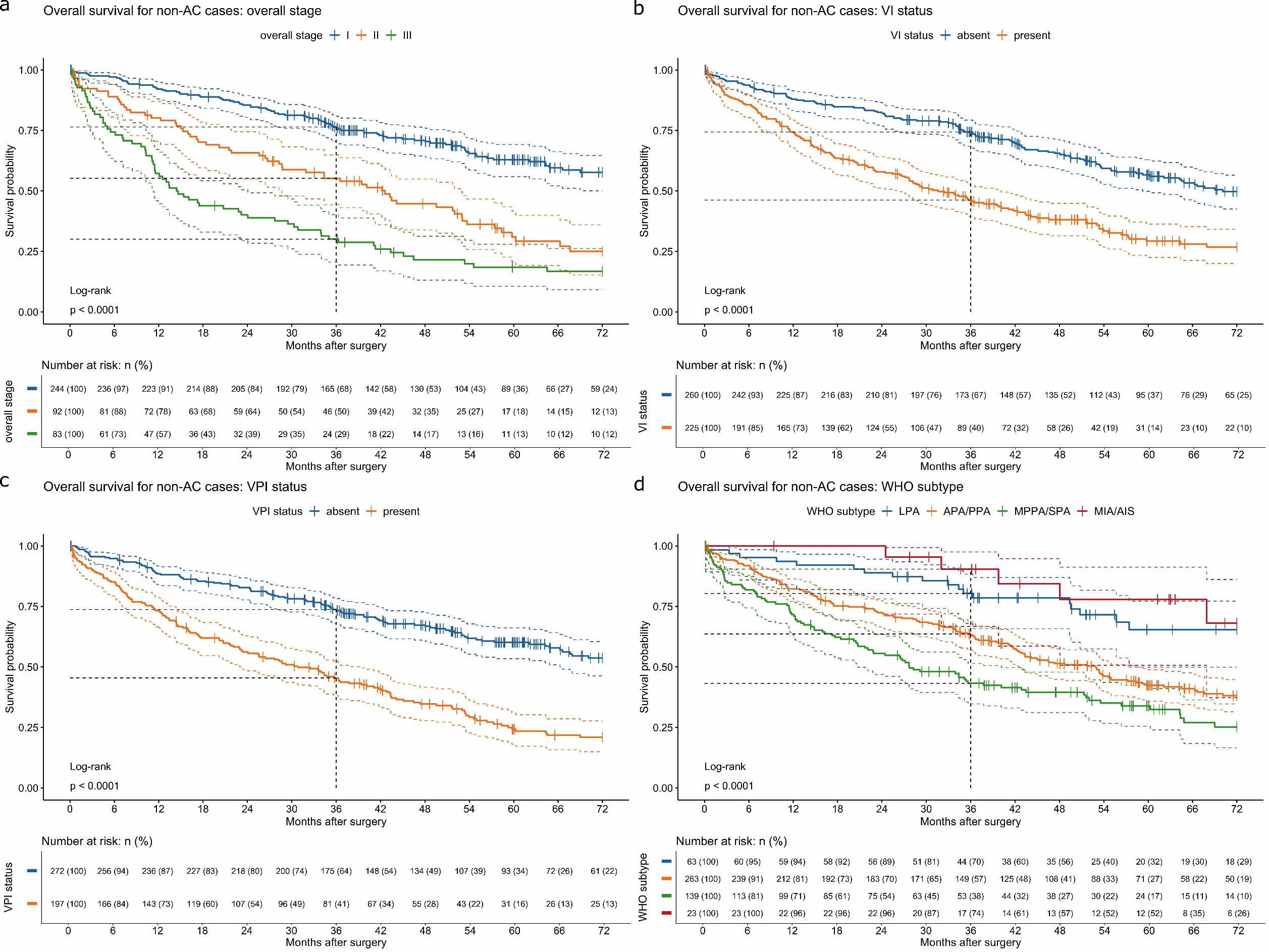


**Notes:**

The patient cohort was divided by a) Overall Stage (TNM 8^th^ edition); b) Vascular invasion status. Significance was assessed by log-rank test.

**Figure S2 Continued: Kaplan-Meier analyses of overall survival by simplified pathological subgroups in patients who did not receive AC**


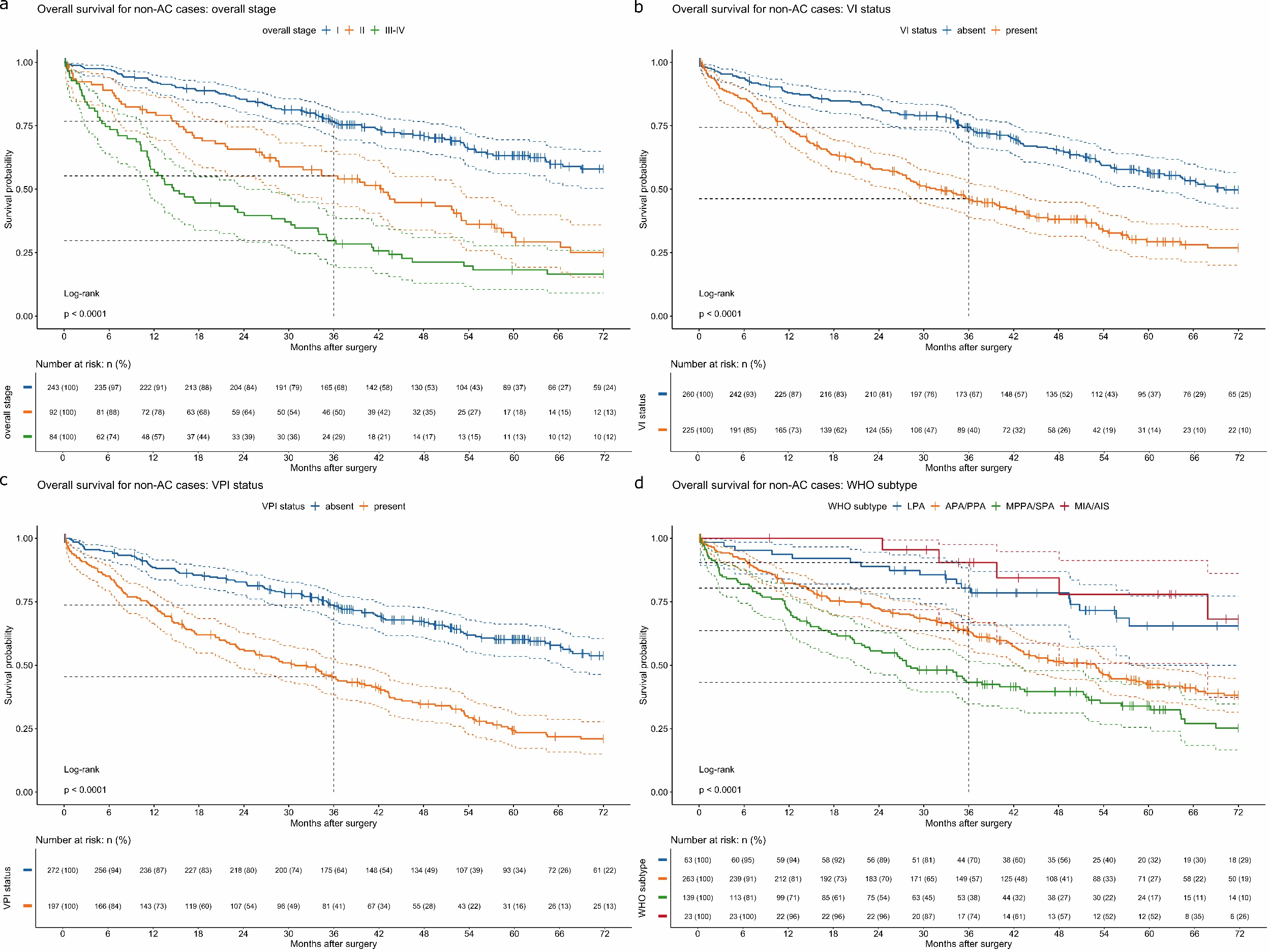


**Notes:**

The patient cohort was divided by c) Visceral pleural invasion status; d) WHO subtype ^a^. Significance was assessed by log-rank test.

**^a^** – LPA: Lepidic adenocarcinoma; APA: Acinar adenocarcinoma; PPA: Papillary adenocarcinoma; SPA: Solid adenocarcinoma; MPPA: Micropapillary adenocarcinoma; MIA: Minimally invasive adenocarcinoma; AIS: Adenocarcinoma *in-situ*

**Figure S3: Kaplan-Meier analyses of overall survival by detailed pathological subgroups in the entire cohort of 620 patients**


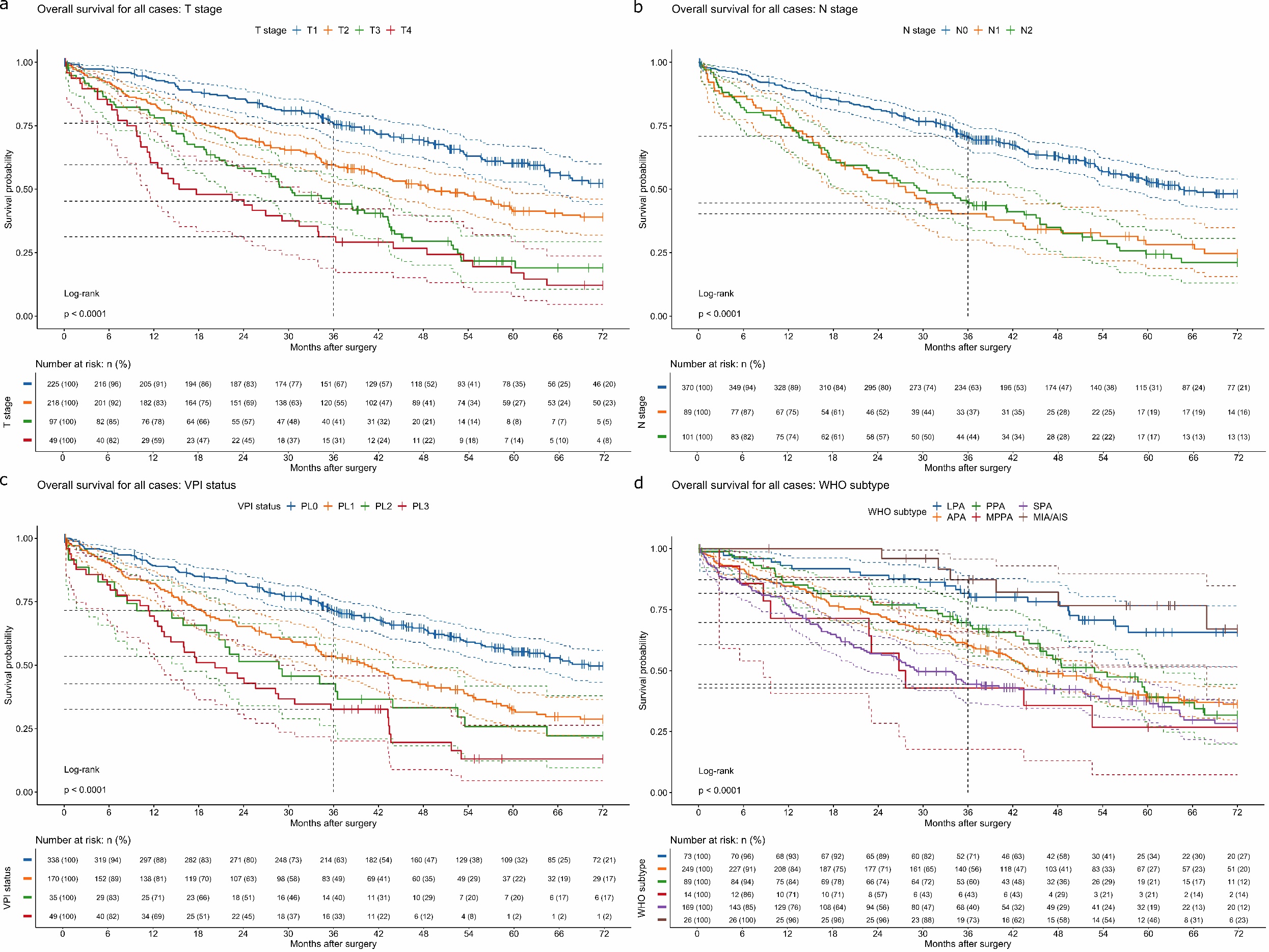


**Notes:**

The patient cohort was divided by a) Pathological T Stage (TNM 8^th^ edition); b) Pathological N Stage (TNM 8^th^ edition). Significance was assessed by log-rank test.

**Figure S3 Continued: Kaplan-Meier analyses of overall survival by detailed pathological subgroups in the entire cohort of 620 patients**


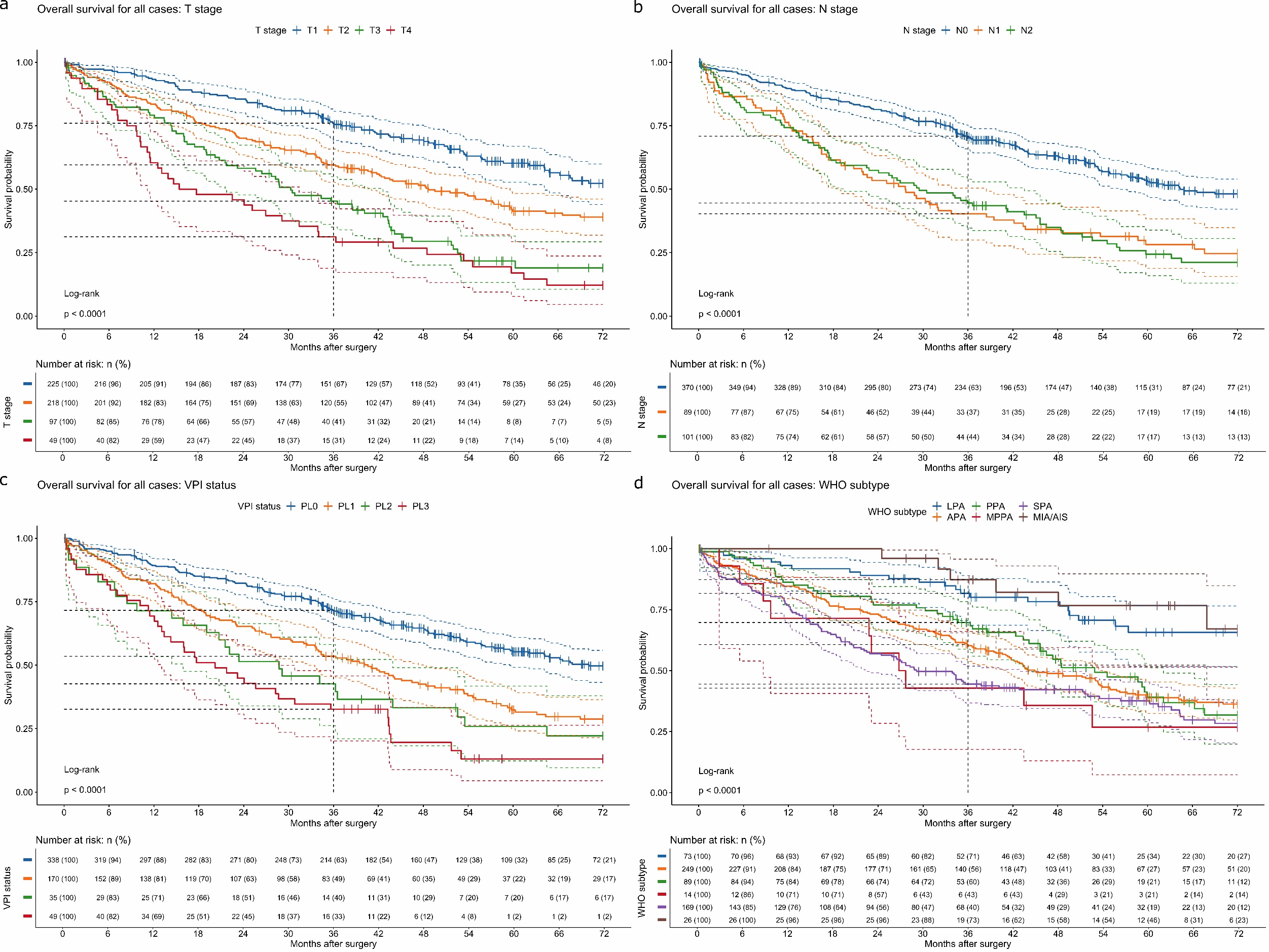


**Notes:**

The patient cohort was divided by c) Visceral pleural invasion status; d) WHO subtype ^a^. Significance was assessed by log-rank test.

**^a^** – LPA: Lepidic adenocarcinoma; APA: Acinar adenocarcinoma; PPA: Papillary adenocarcinoma; SPA: Solid adenocarcinoma; MPPA: Micropapillary adenocarcinoma; MIA: Minimally invasive adenocarcinoma; AIS: Adenocarcinoma *in-situ*

**Figure S4: Kaplan-Meier analyses of overall survival by detailed pathological subgroups in patients who did not receive AC**


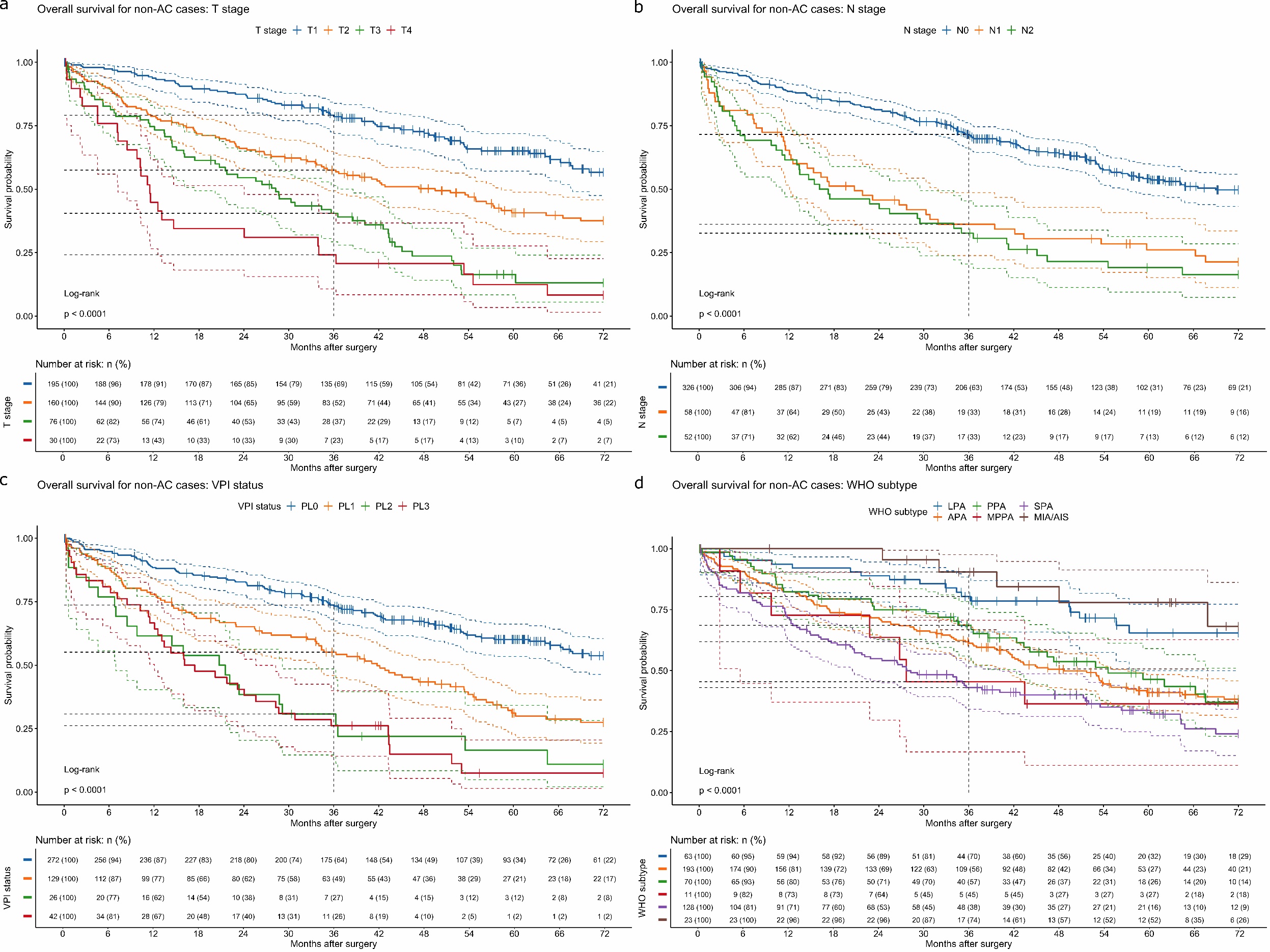


**Notes:**

The patient cohort was divided by a) Pathological T Stage (TNM 8^th^ edition); b) Pathological N Stage (TNM 8^th^ edition). Significance was assessed by log-rank test.

**Figure S4 Continued: Kaplan-Meier analyses of overall survival by detailed pathological subgroups in patients who did not receive AC**


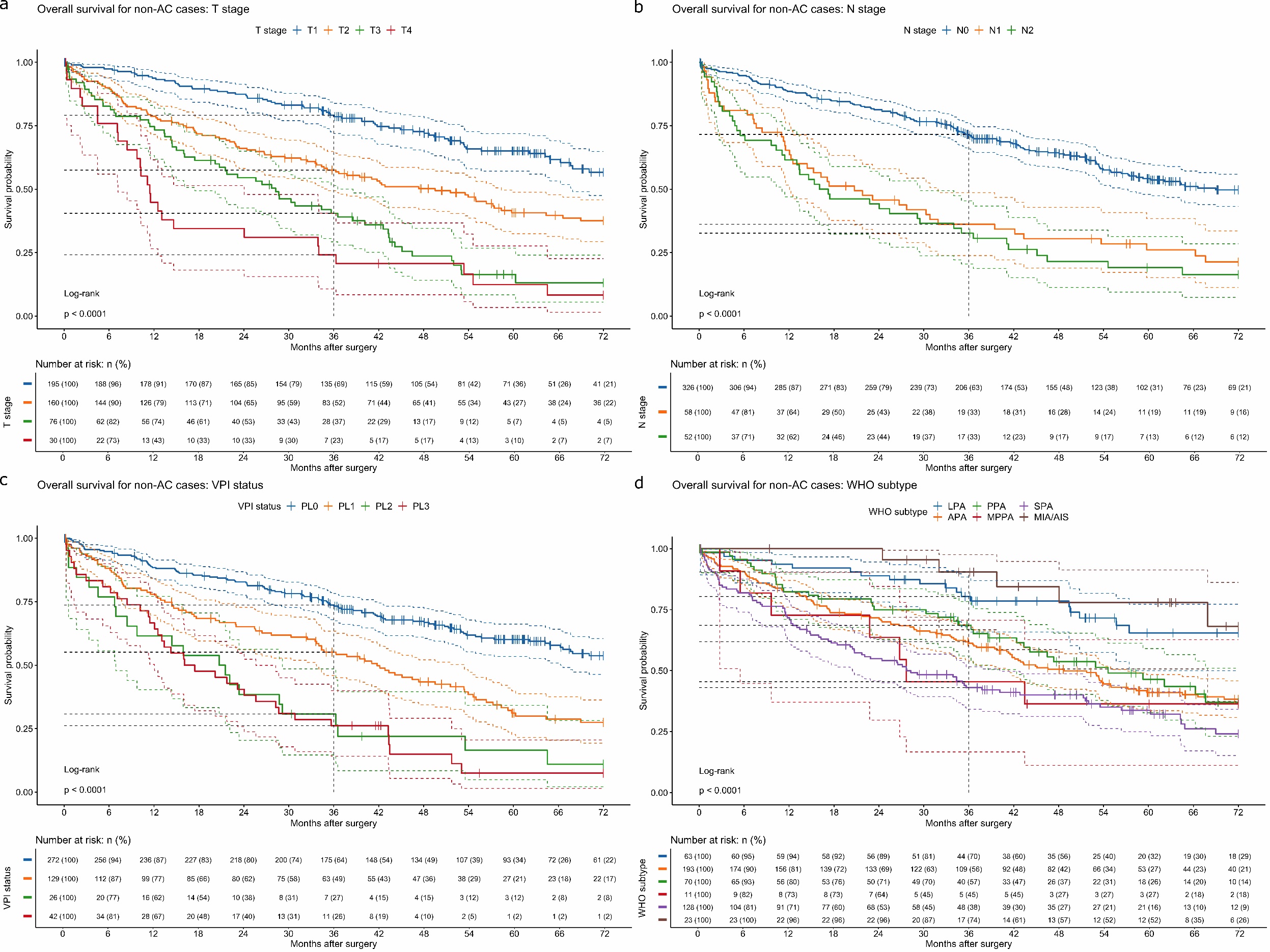


**Notes:**

The patient cohort was divided by c) Visceral pleural invasion status; d) WHO subtype ^a^. Significance was assessed by log-rank test.

**^a^** – LPA: Lepidic adenocarcinoma; APA: Acinar adenocarcinoma; PPA: Papillary adenocarcinoma; SPA: Solid adenocarcinoma; MPPA: Micropapillary adenocarcinoma; MIA: Minimally invasive adenocarcinoma; AIS: Adenocarcinoma *in-situ*

**Figure S5: Unmatched Kaplan-Meier analyses of chemotherapy effects in low- and high-risk pathological subgroups**


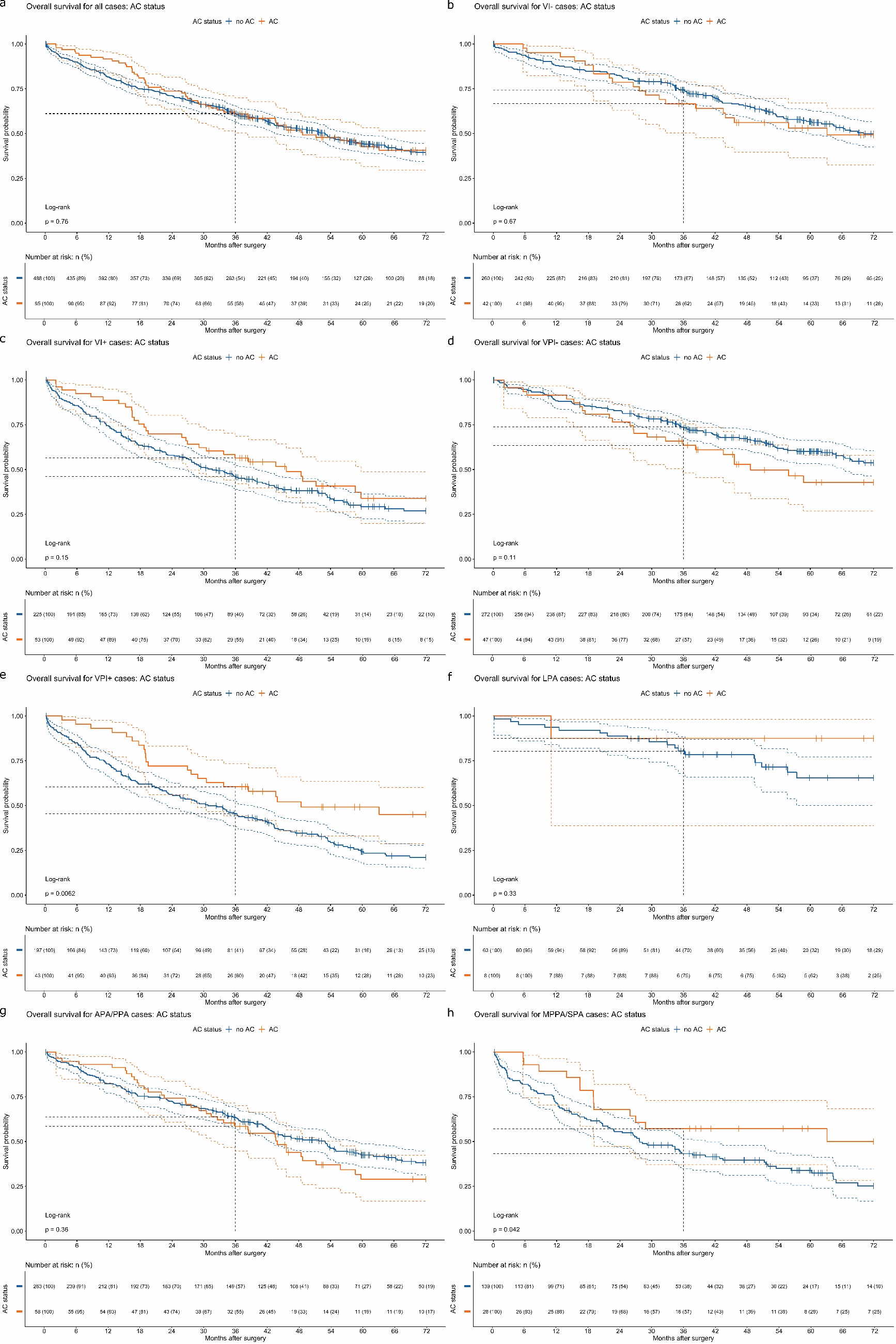


**Notes:**

Unmatched Kaplan-Meier analyses: a) All cases; b) VI- cases ^a^; c) VI+ cases ^b^; d) VPI- cases ^c^. Significance was assessed using log-rank test.

^a^ – Cases with vascular invasion absent

^b^ – Cases with vascular invasion present

^c^ – Cases with visceral pleural invasion absent

**Figure S5 Continued: Unmatched Kaplan-Meier analyses of chemotherapy effects in low- and high-risk pathological subgroups**


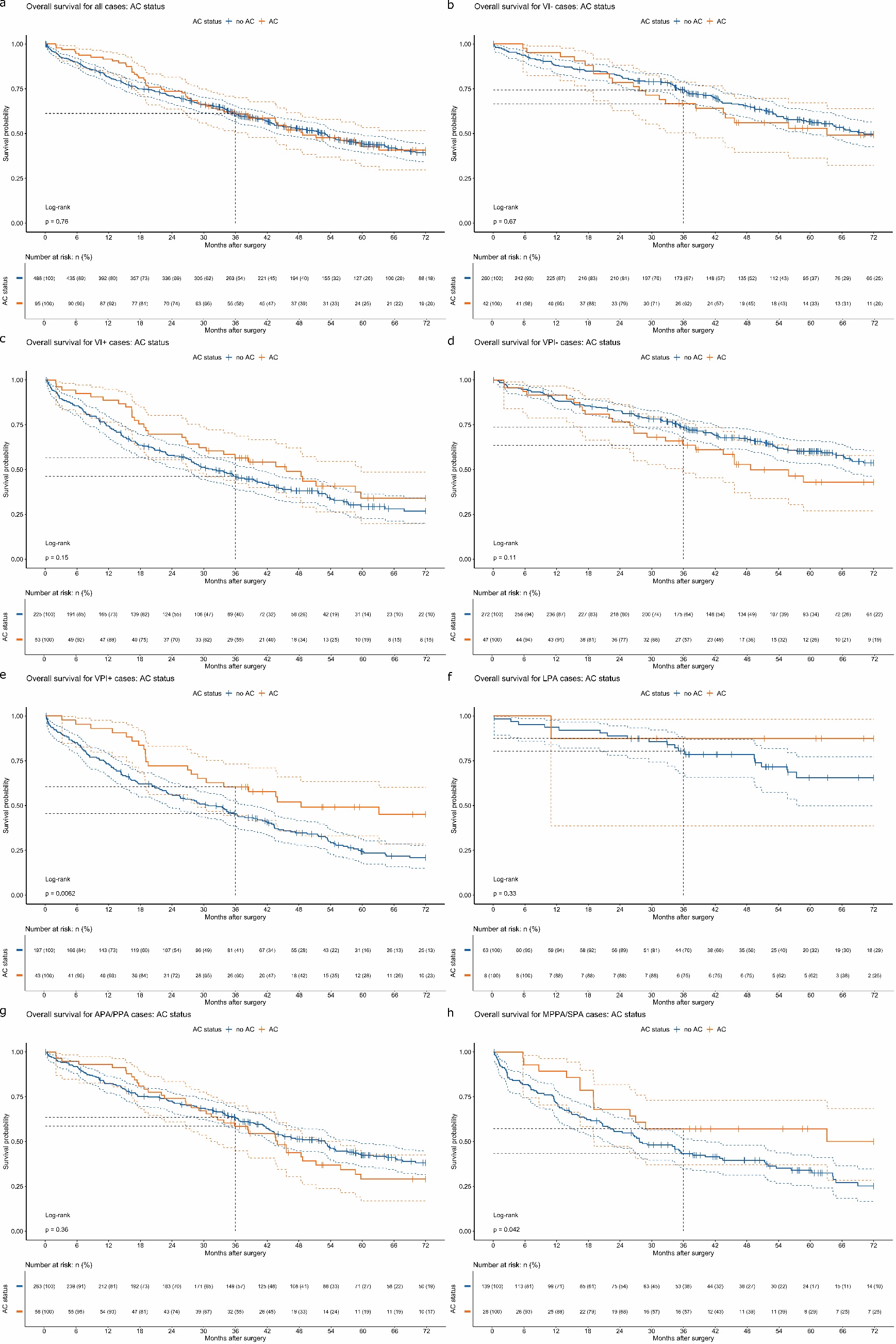


**Notes:**

Unmatched Kaplan-Meier analyses: e) VPI+ cases ^a^; f) WHO subtype LPA cases ^b^; g) WHO subtype APA/PPA cases ^c^; h) WHO subtype MPPA/SPA cases ^d^. Significance was assessed using log-rank test.

^a^ – Cases with visceral pleural invasion present

^b^ – LPA: Lepidic adenocarcinoma

^c^ – APA: Acinar adenocarcinoma or PPA: Papillary adenocarcinoma

^d^ – SPA: Solid adenocarcinoma or MPPA: Micropapillary adenocarcinoma

**Figure S6: Unmatched Kaplan-Meier analyses of patient survival comparing AC- vs non-AC outcomes for existing and augmented sets of AC criteria**


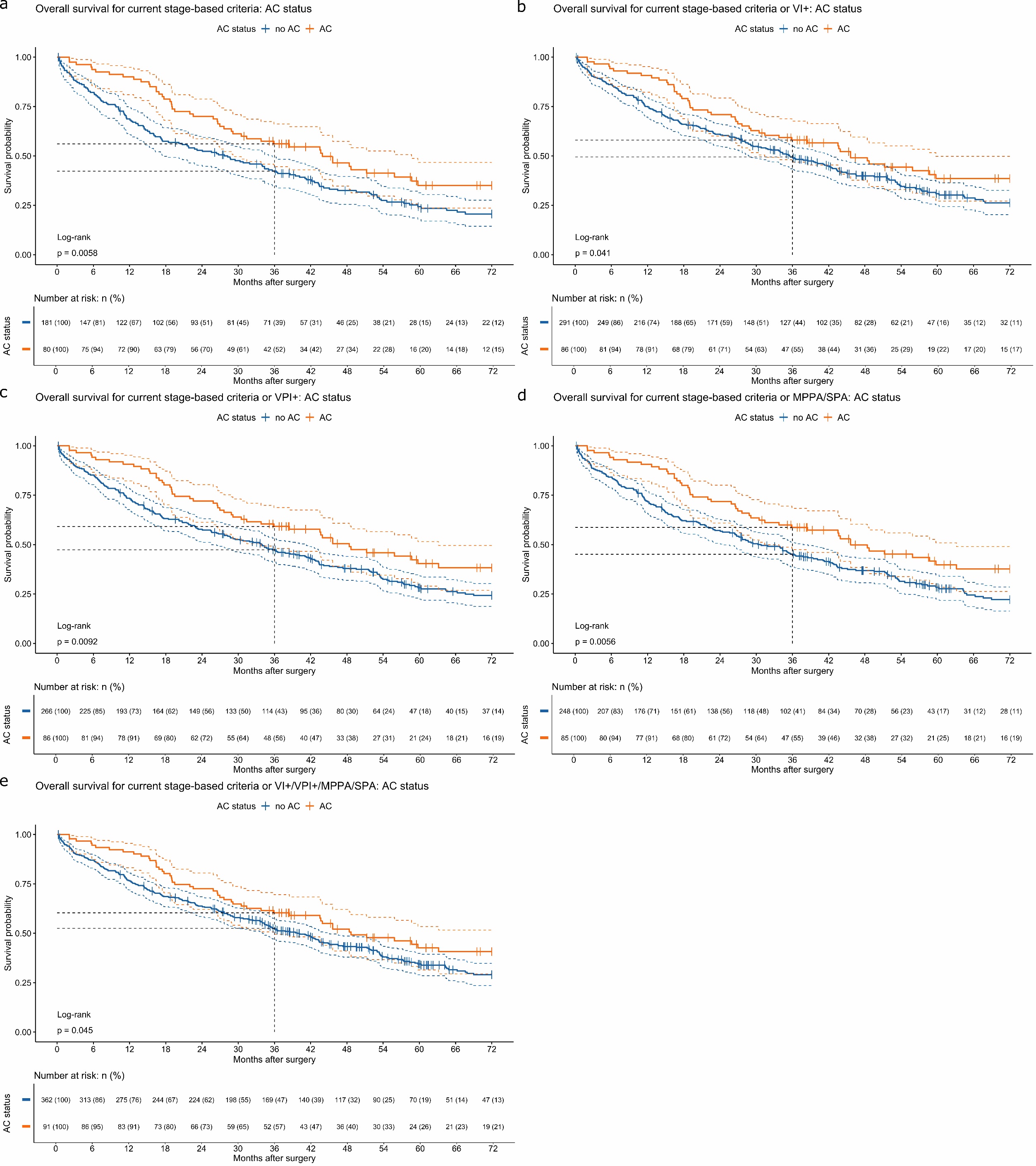


**Notes:**

Unmatched Kaplan-Meier analyses: a) Cases matching current stage-based criteria (>40mm/pN1/pN2 and excluding cases both >50mm/pN2); b) Cases matching current stage-based criteria or with vascular invasion present (VI+). Significance was assessed using log-rank test.

**Figure S6 Continued: Unmatched Kaplan-Meier analyses of patient survival comparing AC- vs non-AC outcomes for existing and augmented sets of AC criteria**


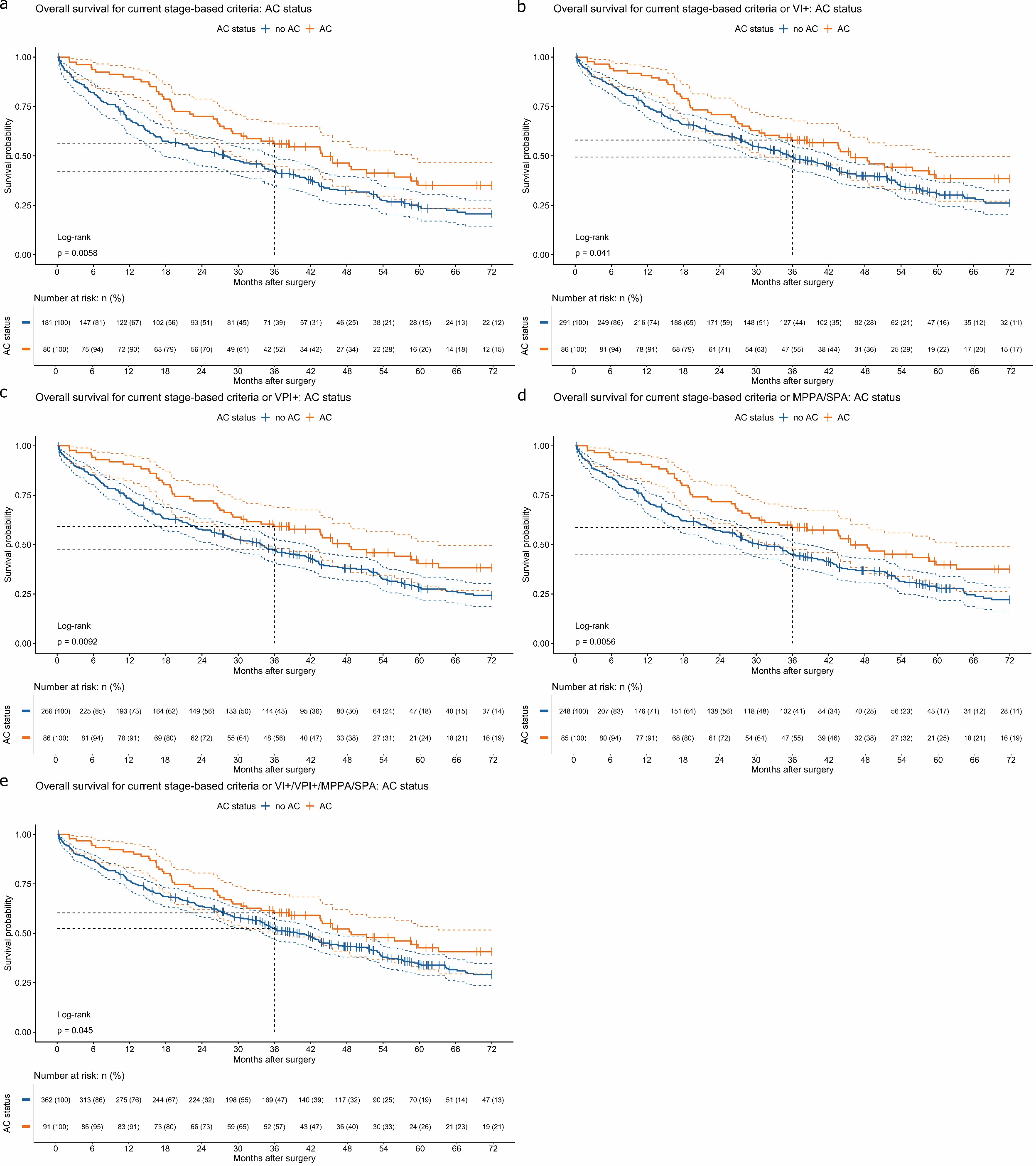


**Notes:**

Unmatched Kaplan-Meier analyses: c) Cases matching current stage-based criteria or with visceral pleural invasion present (VPI+); d) Cases matching current stage-based criteria or with solid adenocarcinoma (SPA)/micropapillary adenocarcinoma (MPPA) WHO subtypes. Significance was assessed using log-rank test.

**Figure S6 Continued: Unmatched Kaplan-Meier analyses of patient survival comparing AC- vs non-AC outcomes for existing and augmented sets of AC criteria**


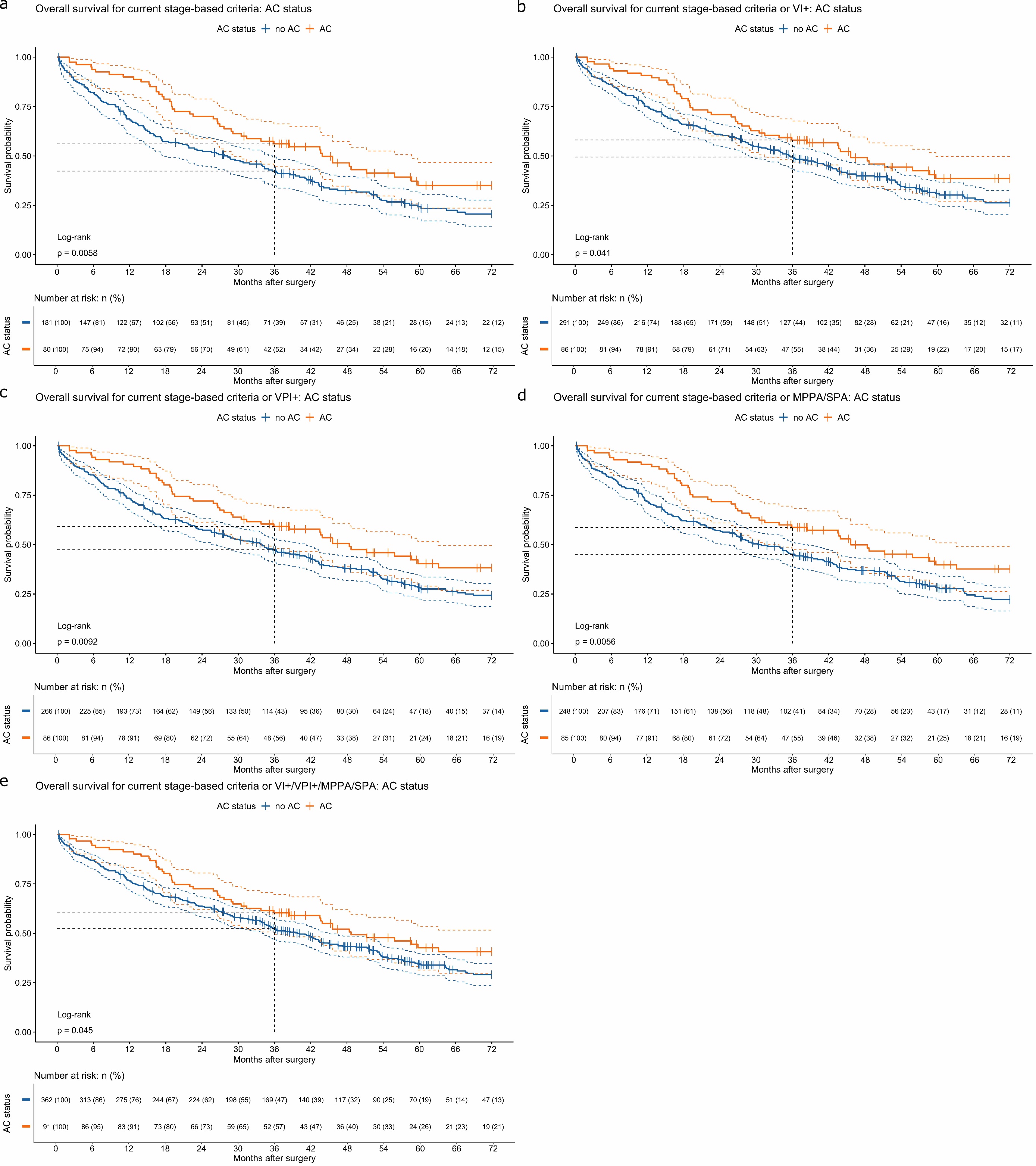


**Notes:**

Unmatched Kaplan-Meier analyses: e) Cases matching current stage-based criteria or with additional VI+/VPI+/SPA/MPPA criteria. Significance was assessed using log-rank test.

**Figure S7: Matched Kaplan-Meier analysis of chemotherapy effects in cases showing high-risk histopathological features (VI+/VPI+/SPA/MPPA) but not meeting current stage-based criteria.**


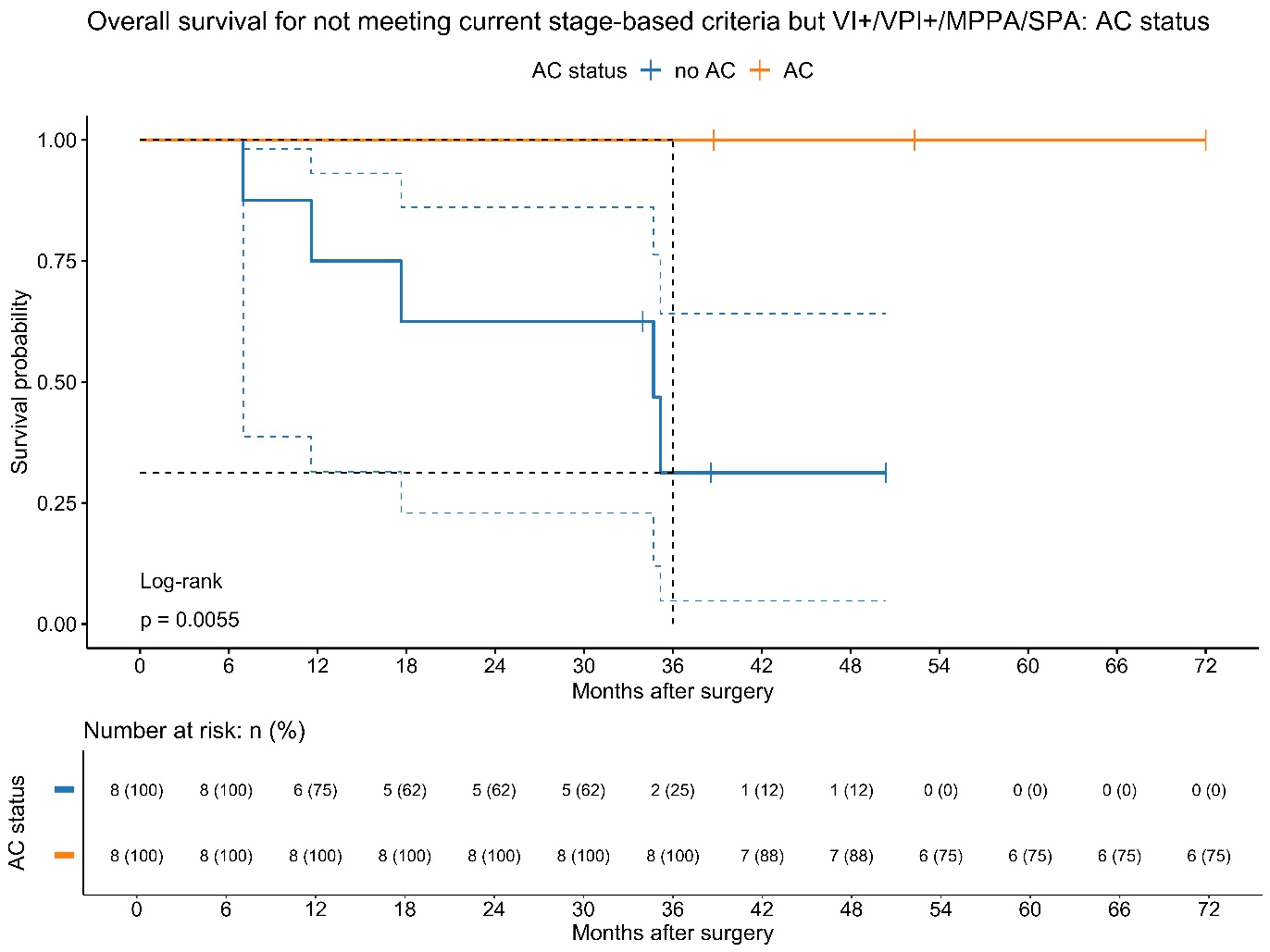


**Notes:**

VI+: Vascular invasion present; VPI+: Visceral pleural invasion present; SPA: Solid adenocarcinoma WHO subtype; MPPA: Micropapillary adenocarcinoma WHO subtype.

Significance was assessed using log-rank test. AC-treated cases were matched to untreated cases by propensity score matching methods.

**Figure S8: Unmatched Kaplan-Meier analysis of chemotherapy effects in cases showing high-risk histopathological features (VI+/VPI+/SPA/MPPA) but not meeting current stage-based criteria.**


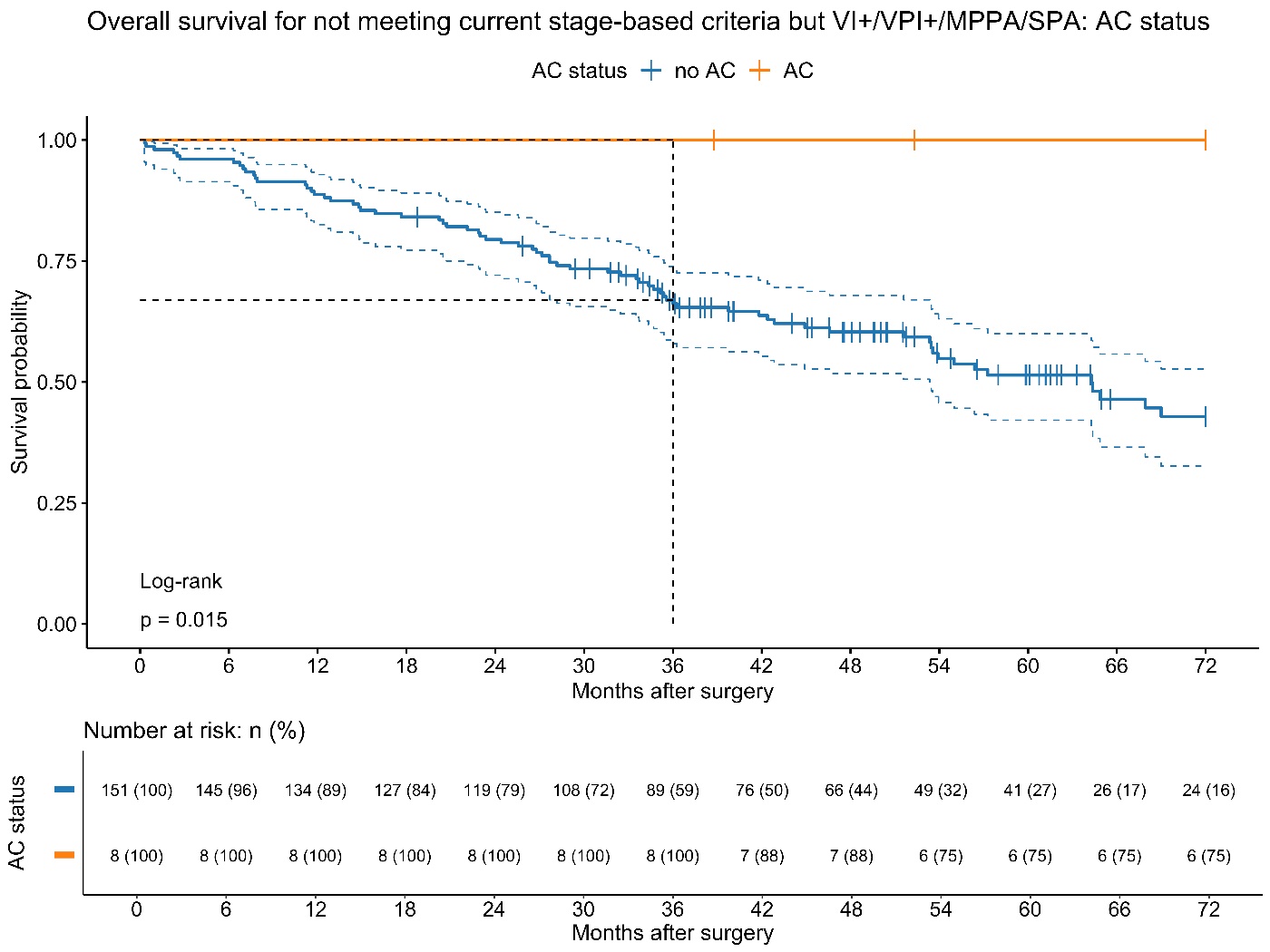


**Notes:**

VI+: Vascular invasion present; VPI+: Visceral pleural invasion present; SPA: Solid adenocarcinoma WHO subtype; MPPA: Micropapillary adenocarcinoma WHO subtype.

Significance was assessed using log-rank test.

**Figure S9: Comparison of the proportion of non-mucinous lung adenocarcinoma cases meeting existing and augmented criteria for the consideration of AC.**


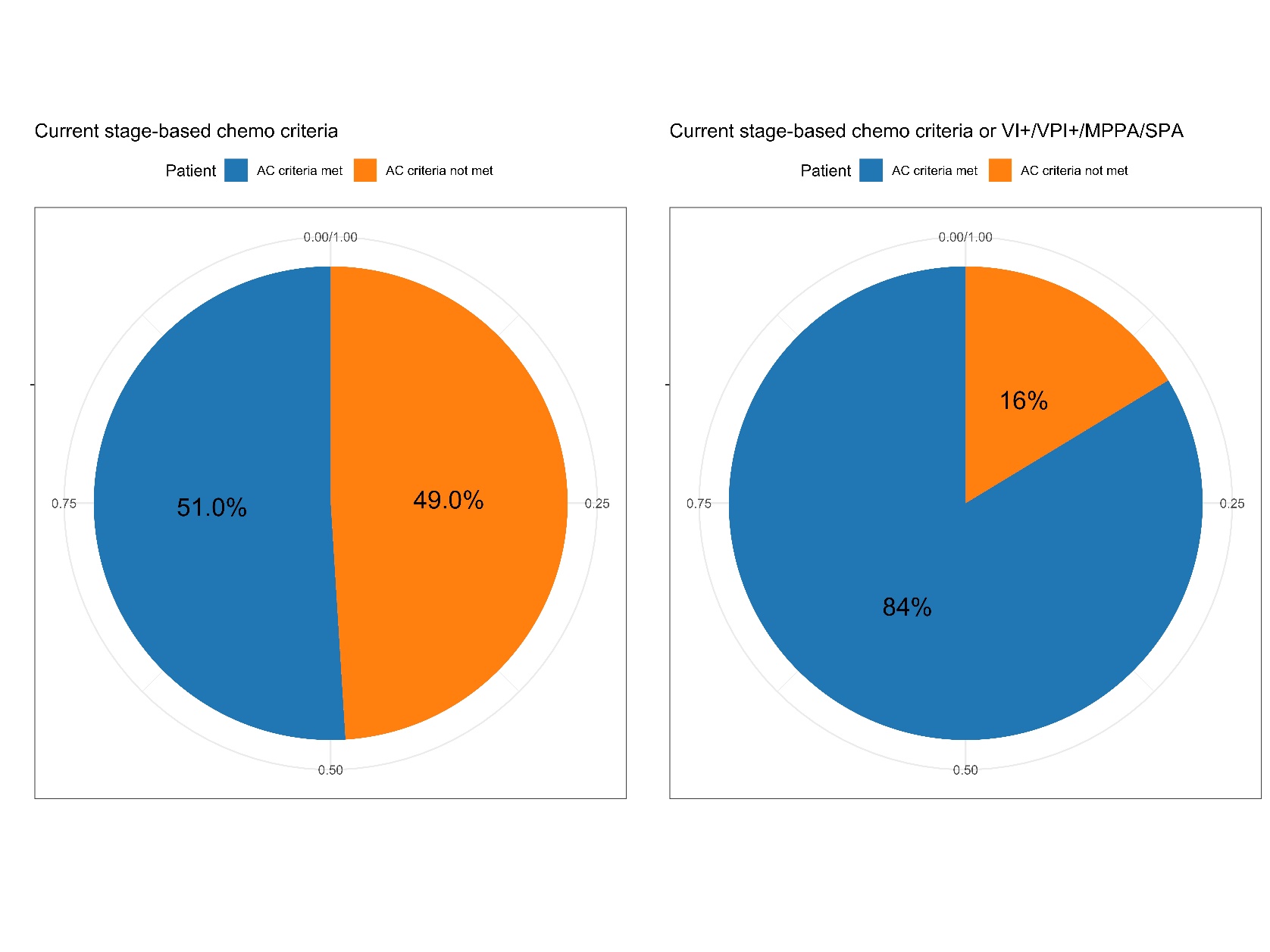


**Notes:**

Current stage-based criteria defined as >40mm/pN1/pN2 but excluding cases which were both >50mm/pN2.

Augmented criteria defined as current stage-based criteria or / VI+/VPI+/SPA/MPPA criteria.

VI+: Vascular invasion present; VPI+: Visceral pleural invasion present; SPA: Solid adenocarcinoma WHO subtype; MPPA: Micropapillary adenocarcinoma WHO subtype.

Significance was assessed using log-rank test.
